# Fingerprinting fragments of fragile interstellar molecules: dissociation chemistry of pyridine and benzonitrile revealed by infrared spectroscopy and theory†

**DOI:** 10.1039/d3fd00015j

**Published:** 2023-03-22

**Authors:** Daniël B. Rap, Aude Simon, Kim Steenbakkers, Johanna G. M. Schrauwen, Britta Redlich, Sandra Brünken

**Affiliations:** a Institute for Molecules and Materials, FELIX Laboratory, Radboud University Toernooiveld 7 6525 ED Nijmegen The Netherlands sandra.bruenken@ru.nl; b Laboratoire de Chimie et Physique Quantiques (LCPQ), Fédération FeRMI, CNRS & Université Toulouse III – Paul Sabatier 118 Route de Narbonne 31062 Toulouse France

## Abstract

The cationic fragmentation products in the dissociative ionization of pyridine and benzonitrile have been studied by infrared action spectroscopy in a cryogenic ion trap instrument at the Free-Electron Lasers for Infrared eXperiments (FELIX) Laboratory. A comparison of the experimental vibrational fingerprints of the dominant cationic fragments with those from quantum chemical calculations revealed a diversity of molecular fragment structures. The loss of HCN/HNC is shown to be the major fragmentation channel for both pyridine and benzonitrile. Using the determined structures of the cationic fragments, potential energy surfaces have been calculated to elucidate the nature of the neutral fragment partner. In the fragmentation chemistry of pyridine, multiple non-cyclic structures are formed, whereas the fragmentation of benzonitrile dominantly leads to the formation of cyclic structures. Among the fragments are linear cyano-(di)acetylene˙^+^, methylene-cyclopropene˙^+^ and *o*- and *m*-benzyne˙^+^ structures, the latter possible building blocks in interstellar polycyclic aromatic hydrocarbon (PAH) formation chemistry. Molecular dynamics simulations using density functional based tight binding (MD/DFTB) were performed and used to benchmark and elucidate the different fragmentation pathways based on the experimentally determined structures. The implications of the difference in fragments observed for pyridine and benzonitrile are discussed in an astrochemical context.

## Abstract 2

Fragmentation pathways of benzonitrile and pyridine are elucidated using cryogenic infrared ion spectroscopy, quantum-chemical calculations and molecular dynamics simulations.

## Graphical abstract

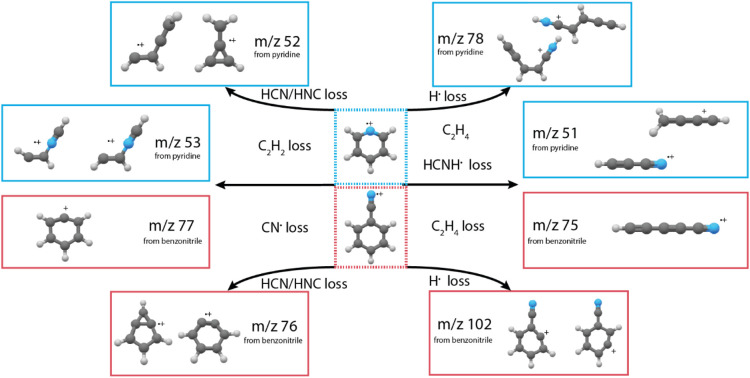

## Introduction

Recently, various aromatic molecules have been detected in the cold interstellar medium (ISM) *via* their rotational transitions in the radio-regime. Among these detections are cyano-substituted aromatic molecules such as cyano-cyclopentadiene,^1,2^ cyano-benzene,^3^ cyano-indene^4^ and cyano-naphthalene^5^ isomers. The discovery of these species has shown that nitrogen can be a part of aromatic molecules and that (cyano)-substitution can be a proxy for the existence of analog pure carbon species.^6^ On the other hand, alternative nitrogen-containing molecules such as pyridine and aniline, also possessing substantial dipole moments, have not been found in the ISM yet despite sensitive searches.^7,8^ Surprisingly, even more complex nitrogen containing molecules such as functionalized pyridines and quinolines have been found in meteorite samples,^9,10^ and have been viewed as possible building blocks of molecules that we consider as necessary for life. Their biological importance lies in the fact that they can be processed to molecules that are part of DNA and RNA similar to life on Earth. Possible so-called bottom-up formation processes towards these larger organic building blocks have been studied using a variety of experimental and theoretical methodologies.^11–17^ However, other processes can counteract these growth mechanisms, *e.g.*, through fragmentation, and can play an important role in the complex interstellar chemistry. These processes can be induced by the presence of high energetic electrons from electromagnetic winds in environments such as Titan’s atmosphere,^18^ or cosmic rays^19^ and ultraviolet (UV) photons^20^ from nearby stars that can induce specific chemistry at the edges of molecular clouds close to photo-dissociation regions (PDRs).^21^ Small carbon-containing molecules have been detected in these regions and are regarded as indirect evidence of top-down hydrocarbon chemistry.^22,23^ Laboratory experiments have shown that both bottom-up and top-down processes can be at play simultaneously. Fragmentation of carbon cycles such as benzene^24,25^ and naphthalene^26,27^ using plasma sources, but also discharges of mixtures of small species such as CH_4_ and N_2_ (ref. 28) have shown that this approach is not always completely destructive, but that recombination chemistry of the fragments and other molecules plays an important role in the formation of larger species. This emphasizes how fragmentation and formation mechanisms are often intertwined.

A variety of studies has been investigating the fragmentation processes of astronomical relevant molecules in detail ranging from theoretical^29–31^ to experimental studies using dissociative ionization and infrared spectroscopy^32–37^ or imaging Photoelectron-Photoion-Coincidence (iPEPICO) spectroscopy.^38–40^ By using a combination of techniques, some of these studies have provided structural information of the fragment ions.

The fragmentation processes of pyridine have been investigated previously using theoretical studies,^41^ electron impact ionization,^42^ VUV photoionization,^43^ kinetic energy release distribution (KERDs) measurements^44,45^ and collision-induced luminescence spectroscopy.^46^ This has shown that the lowest energy fragmentation channel yields ions with the molecular formula C_4_H_4_˙^+^ formed through loss of HCN and/or HNC, which remains the dominant cationic fragment over all ionizing energies.^42,47^ A similar major cationic fragmentation channel was observed in the dissociation of benzonitrile, which has been studied using photodissociation in an ion-cyclotron resonance ion trap,^48^ appearance energy determinations^49,50^ and Rice–Ramsperger–Kassel–Marcus (RRKM) calculations.^51^ These studies pointed toward the formation of the C_6_H_4_˙^+^ fragment upon the loss of HCN and/or HNC. However, no direct structural assignments have been made for these and other cationic fragments of the astrochemically relevant pyridine and benzonitrile molecules to date.

To understand the chemistry in the various environments in space, it is important to have a detailed look at the fragmentation mechanisms of these small nitrogen-containing molecules that have already been detected (benzonitrile) and that are yet unobserved (pyridine). This detailed look should involve accurate structural knowledge of the fragments that, when detected using astronomical measurements, can serve as proxies for the presence of their either undetectable or former parent molecules.

Here we apply a multifaceted approach to investigate the fragmentation chemistry of the nitrogen-containing molecules pyridine and benzonitrile in detail. Combining dissociative ionization and cryogenic infrared pre-dissociation spectroscopy (IRPD) using the free-electron laser FELIX,^52,53^ we measure the infrared fingerprints of the major ionic fragments. The molecular structures of the fragment products are assigned by comparison to quantum-chemical calculations and are used to guide the construction of potential energy surfaces (PESs) that explain the nature of the fragmentation reaction. Finally, we have performed molecular dynamics (MD) simulations with the electronic structure determined at the self-consistent-charge density functional based tight binding (SCC-DFTB)^54^ level of theory to predict the observed fragmentation channels including the molecular structures. Such a combined approach has been successfully used to disentangle the dissociation mechanism of cationic PAHs such as anthracene and phenanthrene.^37^ This work can guide experimental and astronomical studies in finding new astrochemical relevant species, and, furthermore, provides vibrational fingerprint spectra of dominant fragment ions as reference data for sensitive infrared astronomical searches, *e.g.*, with the James Webb Space Telescope (JWST).^55^

## Results and discussion

### Mass spectrometry of pyridine and benzonitrile fragmentation

**Fig. 1:**
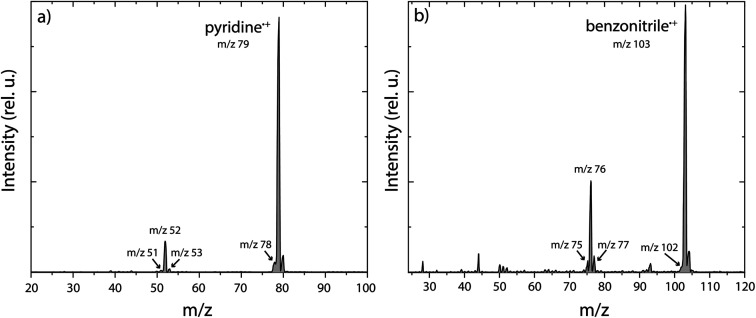
Experimental mass spectra of the dissociative ionization of (a) pyridine and (b) benzonitrile at an electron impact energy of 15 eV. The fragments that are studied in this paper are labelled with their *m*/*z* value.

The experimental and theoretical methods used in the present work are described in more detail in the ESI.† Only the results are reported and discussed in the rest of the manuscript. Main fragmentation channels were identified by measuring the number of ions of the different fragment masses (*m*/*z*) in the dissociative ionization of pyridine and benzonitrile at various electron impact energies, see Supplementary Methods in the ESI† for details of the experimental apparatus. An example breakdown curve for the dissociative ionization of benzonitrile is shown in Supplementary Fig. 1,† and it agrees within our uncertainties with previous electron impact ionization studies. The ionization energies of pyridine and benzonitrile are 9.34 eV (electron impact ionization)^56^ and 9.73 eV (threshold electron detection),^57^ respectively. At low electron impact energies of 15(2) eV, mainly the HCN/HNC fragment channel, with an appearance energy (AE) of 12.34 eV (ref. 58) and 12.54 eV (ref. 50) for pyridine and benzonitrile, respectively, is accessible and other fragment channels are present in minor abundance. This is demonstrated in the mass spectra for both pyridine and benzonitrile as shown in Fig. 1a and b, respectively. Upon higher electron energies (50 eV), we observe more contribution from other energetically higher-lying fragment channels. From the *m*/*z* values we can deduce the possible neutral loss fragments. We observe the loss of a hydrogen atom (H, −1 *m*/*z*, AE = 14 eV for pyridine^47^), acetylene (C_2_H_2_, −26 *m*/*z*, AE = 13.84 eV for pyridine^47^) and/or cyano radical CN˙ (−26 *m*/*z*, AE = 13.52 eV for benzonitrile^58^), hydrogen cyanide and/or hydrogen isocyanide (HCN and/or HNC, −27 *m*/*z*, AE = 12.34 eV (ref. 58) and 12.54 eV (ref. 50) for pyridine and benzonitrile, respectively) and ethylene and/or dihydrogen cyanide (C_2_H_4_ and/or H_2_CN˙, −28 *m*/*z*, AE = 16.6 eV for pyridine^47^) for both parent molecules.

**Fig. 2:**
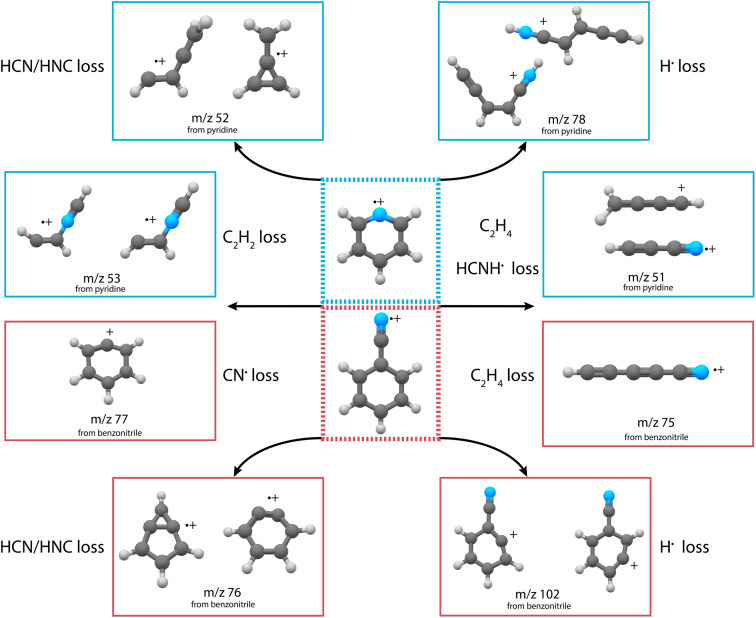
Schematic overview of the discovered structures from the major fragmentation channels upon dissociative ionization of pyridine (blue boxes) and benzonitrile (red boxes).

For both pyridine and benzonitrile, the most abundant fragment, *m*/*z* 52 and *m*/*z* 76, is formed upon the loss of either HCN or HNC. However, based on the observed mass difference alone, the exact mechanism cannot be deduced as structurally different neutral and cationic fragment molecules can be formed. Looking at the nature of the cationic fragment species can help to understand the fragmentation mechanism. In order to determine the molecular structure of the observed fragment ions, we have performed infrared pre-dissociation spectroscopy using the free-electron laser FELIX.^52^ The fragments that were produced in the ion source were mass-selected and their infrared fingerprint were measured in the 550–2200 cm^−1^ range (see Supplementary Methods† for details of the experimental setup). Here, the HCN/HNC loss fragments were produced using electron impact energies of 15(2) eV; however, to get a sufficient number of ions to probe the minor fragmentation channels, an electron energy of 50(2) eV was used. Comparing the recorded fingerprint spectra to calculated infrared vibrational modes using density functional theory (DFT) allows assignment of molecular structures to the fragment ions, see Supplementary Methods† for details of these calculations. The major fragmentation channels and their accompanying molecular structures that have been identified in this way for both the dissociative ionization of pyridine and benzonitrile are discussed throughout the paper and are schematically shown in Fig. 2.

### Spectroscopy of pyridine^+^ fragmentation products

**Fig. 3:**
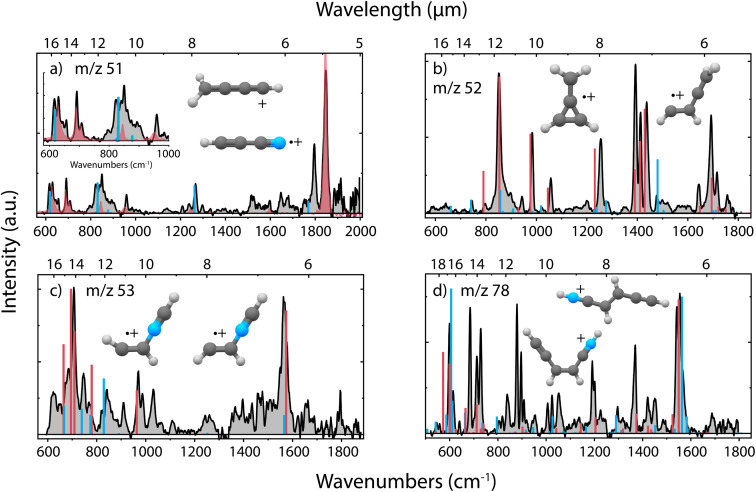
Experimental infrared fingerprint spectra (grey) of (a) *m*/*z* 51, (b) *m*/*z* 52, (c) *m*/*z* 53 and (d) *m*/*z* 78 fragment channels of the dissociative ionization of pyridine. For each panel, the *x*-axis is showing wavenumbers in cm^−1^ (lower axis) and wavelength in μm (upper axis). An experimental reference spectrum of cyanoacetylene˙^+^ (HC_3_N˙^+^) (red trace) from Steenbakkers *et al.*^59^ is shown in panel (a). Calculated vibrational modes of the assigned structures are shown for (a) protonated diacetylene^+^ (l-CH_2_CCCH^+^) (blue), (b) methylene-cyclopropene˙^+^ (c-C_3_H_2_(CH_2_)˙^+^) (red), CHCHCCH_2_˙^+^ (blue), (c) protonated *trans*-isocyanoacetylene^+^ (*trans*-CHCHNCH^+^) (red), protonated *cis*-isocyanoacetylene^+^ (*cis*-CHCHNCH^+^) (blue) and (d) *cis*-CHCCHCHCNH^+^ (red), *trans*-CHCCHCHCNH^+^ (blue) as sticks. The infrared spectra have been calculated at the harmonic B3LYP-GD3/N07D level of theory. The anharmonic spectra of methylene-cyclopropene˙^+^ (*m*/*z* 52) and *cis*/*trans* CHCCHCHCNH^+^ (*m*/*z* 78) have been used to improve the match with the experimental spectrum due to the calculated strong combination and overtone modes. The spectrum of protonated diacetylene (*m*/*z* 51) has been calculated at the B2PLYPD3/aug-cc-pVTZ level of theory.

Upon dissociative ionization of pyridine, we observe the formation of cationic fragments with *m*/*z* 51, 52, 53 and 78. For each of the fragment channels, the recorded infrared fingerprint spectrum is shown in Fig. 3 together with the calculated or previously measured infrared spectra and the structures of the assigned molecules. Multiple additional candidate structures have been evaluated and optimized, and their infrared spectra have been calculated as described in the Supplementary Methods and are shown in the ESI (Supplementary Fig. 3 and 7–9),† but do not show a sufficient match with the experimental data.

The major fragmentation channel (*m*/*z* 52) can be assigned to the lowest energy isomeric methylene-cyclopropene˙^+^ structure containing a 3-membered ring. A minor contribution of a non-cyclic CHCHCCH_2_˙^+^ species, hinting to a ring-opening process of methylene-cyclopropene˙^+^, is observed. Using a saturation depletion experiment, the abundance of the major isomer is estimated to be around 85–90%. The other isomer is measured to account for 15–20% (more details in Supplementary Fig. 5 and 6†). This non-cyclic structure is significantly higher in energy (130.7 kJ mol^−1^) than the other isomers butatriene˙^+^ (9.7 kJ mol^−1^) or cyclobutadiene˙^+^ (39.9 kJ mol^−1^) (Supplementary Fig. 3†), with the latter being characterized previously by Ar-IRPD spectroscopy.^60^ This hints to some excess energy in the ion that enables further ring opening, for which a barrier of 174 kJ mol^−1^ (1.8 eV) (at the MP2/6-311++G(d,p) level of theory) has been calculated.^61^ For the other fragmentation channels, we generally observe non-cyclic compounds.

The *m*/*z* 78 fragment can be assigned to the H-loss fragments from cationic pyridine where a hydrogen is migrated to the nitrogen atom and the aromatic ring has opened. The spectrum can be assigned to the *cis* and *trans* isomers that have similar energies, based on the double peak structure around 1550 cm^−1^, related to the C=C double bond stretching vibration from each of the two isomers. Small frequency shifts between the calculated and experimental spectra are observed between 550 and 750 cm^−1^ due to anharmonicity effects and are hampering a clear assignment. To validate the structural assignment, the infrared spectrum in the N–H stretch region was measured using a table-top OPO/OPA system and showed two features, presenting both the NH and CH stretching modes, thereby confirming the presence of an N–H group in the molecule (Supplementary Fig. 10†). Both assigned species are 43.2 and 41.3 kJ mol^−1^ higher in energy than the global minimum structure, the cyclic 1-dehydro-pyridine˙^+^ (Supplementary Fig. 7†), where a hydrogen has been removed on the C1 position. This demonstrates that under the experimental conditions (EI of 50 eV, single collision conditions) a hydrogen migration towards the nitrogen and subsequent ring opening is more probable than direct H-loss from the ring. Clear evidence of H-migration to the nitrogen atom before dissociation of pyridine molecules has been discussed previously by Wasowicz *et al.*^62,63^

To elucidate the structure of the *m*/*z* 53 fragment, many potential species with the molecular formula C_3_H_3_N^+^ as discussed by Bera *et al.*^64^ have been considered (Supplementary Fig. 8†). Two isomers, protonated *cis*- and *trans*-isocyanoacetylene^+^, can be assigned and contain a diagnostic C=C double bond stretching mode around 1565 cm^−1^, similar as for the H-loss species. A few experimental bands have not been assigned but can come from anharmonic contributions such as overtones and combination bands. The assignment of these N-containing species for *m*/*z* 53 confirms the neutral fragment to be C_2_H_2_ rather than the loss of CN, which would lead to the C_4_H_5_^+^ ionic fragment, whose lowest energy isomer is the methyl-cyclopropyl cation, for which high-level vibrational frequency calculations exist.^65^ The observed species are not the global minimum on the C_3_H_3_N^+^ PES, which has a CCCN backbone geometry as calculated by Bera *et al.*,^64^ but they have been proposed as one of the intermediates in the C_2_H_2_^+^ + HCN ion–molecule reaction.^66^

The major fragment in the *m*/*z* 51 channel is assigned to cyanoacetylene˙^+^ (HC_3_N˙^+^) where a neutral C_2_H_4_ fragment is formed. This open-shell linear ion exhibits a ^2^Π electronic ground state and is therefore subjected to Renner–Teller vibronic coupling effects. The spectrum of this ion has been recently recorded in our group by means of Ne-tagging IRPD,^59^ where it was produced from the vinyl cyanide neutral precursor, and we use it here as a reference spectrum to assign the fragment ion structure. We may see some contribution of isocyanoacetylene˙^+^, based on the slightly shifted experimental band around 1795 cm^−1^ due to the CC stretching vibration of the CNC bond. This open-shell linear ion is also Renner–Teller affected and we have constructed an effective Hamiltonian for the bending vibrations using harmonic frequencies and spin–orbit parameters calculated using the RCCSD(T)-F12a/cc-pVTZ-F12 level of theory (Supplementary Fig. 9†). This allows the making of a comparison with similarly determined frequencies of the affected bending modes of cyanoacetylene˙^+^ (Supplementary Fig. 9, see also Supplementary Methods†). All bending modes of isocyanoacetylene˙^+^ are shifted to lower frequencies and do not fully explain the additional experimental bands in the range below 900 cm^−1^. However, the other vibrational modes, both in the range lower than 900 cm^−1^ and the feature around 1265 cm^−1^, can be assigned to protonated diacetylene which is formed by the loss of a species with molecular formula H_2_CN˙. The cationic fragment has been calculated to be the global minimum on the C_4_H_3_^+^ PES, with an analogous structure to the assigned *m*/*z* 52 fragment, c-C_3_H_2_(CH)^+^, lying 89 kJ mol^−1^ higher in energy (Supplementary Fig. 9†). Whereas the electronic spectrum of protonated diacetylene is known,^67,68^ only a limited range of the infrared spectrum was covered in a neon matrix spectroscopy study.^67^ This all leads to the conclusion that we observe the presence of both cyanoacetylene˙^+^ and protonated diacetylene^+^, but we cannot exclude some additional contribution of isocyanoacetylene˙^+^.

### Spectroscopy of benzonitrile^+^ fragmentation products

**Fig. 4:**
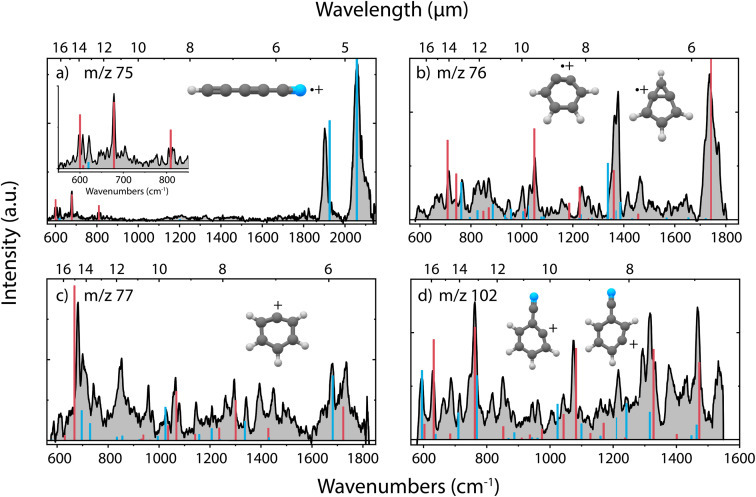
Experimental infrared fingerprint spectra (grey) of (a) *m*/*z* 75, (b) *m*/*z* 76, (c) *m*/*z* 77 and (d) *m*/*z* 102 fragment channels of the dissociative ionization of benzonitrile. For each panel, the *x*-axis is showing wavenumbers in cm^−1^ (lower axis) and wavelength in μm (upper axis). Calculated vibrational modes are shown for (a) cyano-diacetylene˙^+^ (HC_5_N˙^+^) (red/blue), (b) *o*-benzyne˙^+^ (C_6_H_4_˙^+^) (red), bicyclic *m*-benzyne˙^+^ (C_6_H_4_˙^+^) (blue), (c) phenyl^+^ (C_6_H_5_^+^) (red), ^13^C-*o*-benzyne˙^+^ (^13^CC_5_H_4_˙^+^) (blue) and (d) 2-dehydro (C_7_H_4_N^+^) (blue) and 3-dehydro-benzonitrile^+^ (C_7_H_4_N^+^) (red) as sticks. The infrared spectra have been calculated at the harmonic B3LYP-GD3/N07D level of theory. The anharmonic infrared spectrum of bicyclic *m*-benzyne˙^+^ (*m*/*z* 76) has been calculated to improve the match with the experimental spectrum. The Renner–Teller affected bending modes of HC_5_N˙^+^ (red, panel (a)) have been determined using an effective Hamiltonian approach and are combined with (calculated) harmonic stretching modes (blue) by Gans *et al.*^69^

In the dissociative ionization of benzonitrile, we observe the formation of *m*/*z* 75, 76, 77 and 102, corresponding to similar fragmentation channels as for pyridine, namely C_2_H_4_/H_2_CN˙, HCN/HNC, C_2_H_2_/CN˙ and H-loss, respectively. The experimental infrared fingerprint spectra of these fragments are shown in Fig. 4, together with the calculated infrared spectra of the assigned molecular structures. The infrared frequencies of multiple additional potential structures were also calculated and are shown in Supplementary Fig. 13–16.†

Generally, we observe more cyclic structures across the fragment channels than for the pyridine fragmentation. The molecular structures of the most abundant fragment with *m*/*z* 76 can be assigned to a combination of two isomers, *i.e.*, the *ortho* (*o*) and a bicyclic *meta* (*m*)-benzyne˙^+^ species. The characteristic CC stretch of *o*-benzyne˙^+^ is calculated around 1740 cm^−1^ and is experimentally observed. The structure of *o*-benzyne˙^+^ shows a deformation of the ring making the structure non-planar, which has been previously observed by Kaiser *et al.*^70^ using photoelectron spectroscopy. No saturation depletion measurements have been performed due to experimental difficulties, but the recorded relative depletion of the strong and unique 1740 cm^−1^ stretching band can be regarded as a lower limit for the *o*-benzyne˙^+^ abundance, indicating the abundance to be larger than 65%.

To confirm the assignment of the other isomer, we have measured the experimental infrared spectrum of the H_2_ loss fragment of cationic benzene, as for neutral benzene dissociation it has been calculated that neutral (*o*/*m*/*p*)-benzyne isomers can be formed^71^ (Supplementary Fig. 11†). Comparison with anharmonic infrared frequency calculations of both *m*-benzyne˙^+^ and a more bicyclic *m*-benzyne˙^+^ species shows clear evidence of the existence of the latter. Comparing these reference frequencies to the spectrum of *m*/*z* 76 from benzonitrile confirms the presence of this bicyclic *m*-benzyne˙^+^ structure as the other minor fragment (Supplementary Fig. 12†). The most important difference between the two *meta* isomers is that their CC bond lengths differ between 1.968 Å and 1.422 Å (at the B3LYP-GD3/N07D level of theory) for the *m*-benzyne˙^+^ and bicyclic *m*-benzyne˙^+^, respectively, which changes the infrared spectrum significantly and makes them spectroscopically distinguishable. While for neutral *m*-benzyne the biradicaloid structure is favored to the closed shell bicyclic structure,^72^ we observe that for the radical cation the bicyclic structure exists as a stable fragmentation product of benzonitrile.

Two dehydro-benzonitrile^+^ isomers are assigned as the H-loss fragments with *m*/*z* 102 indicating that direct H-loss processes take place without ring opening. This contrasts with the pyridine H-loss channel where two non-cyclic structures are observed. Based on the calculated vibrational modes, we observe predominantly the 2- and 3-dehydro isomers where the hydrogen is lost at the C2 and C3 carbon atom, respectively (Supplementary Fig. 14†). The 3- and 4-dehydro isomers are both similar in energy and the lowest for this channel, however, calculated modes of the latter do not contribute significantly to the experimental spectrum.

In the experimental spectrum of the *m*/*z* 77 fragment, CN˙- or C_2_H_2_-loss, two features around 1680 and 1735 cm^−1^ are observed, indicating two structures with different CC stretching vibrations. The 1735 cm^−1^ band and many other features can be associated with the phenyl^+^ structure. This assignment is strengthened by the comparison with experimental infrared frequencies from Wiersma *et al.*^73^ The result shows that we observe the loss of CN˙, rather than the loss of C_2_H_2_. The other CC stretching mode around 1680 cm^−1^ is likely due to an interesting structure containing a CH group and a 5-membered ring that was observed at the B3LYP-GD3/N07D level of theory (Supplementary Fig. 15†). However, we are more confident to assign this mode to some contribution of the ^13^C isotopologues of the abundant *o*-benzyne˙^+^ fragment that overlaps in this mass channel. Interestingly, also for protonated benzonitrile^+^, the major fragmentation channel, which is HCN/HNC loss, leads to the formation of phenyl^+^.^74^

The smallest fragment with *m*/*z* 75 can be assigned to cyanodiacetylene˙^+^ (HC_5_N˙^+^), a structure similar to cyanoacetylene˙^+^ as the fragment from pyridine, where in both systems neutral C_2_H_4_ is lost. This open-shell linear ion is again Renner–Teller affected, but to our knowledge its vibrational spectrum has not been recorded previously. The assignment was therefore based on a combination of harmonic vibrational frequencies for the stretching modes and an effective Hamiltonian approach for the Renner–Teller affected bending modes. Both the harmonic frequencies and the Renner–Teller and spin–orbit parameters were taken from Gans *et al.*^69^ (calculated at ic-MRCI+Q/AVTZ level of theory). The intensities here were calculated at the B3LYP-GD3/N07D level of theory. We see no trace of pure carbon species such as cyclic C_6_H_3_^+^ which is calculated to be stable but difficult to observe experimentally^75^ (Supplementary Fig. 16†).

### Potential energy surfaces of HCN/HNC loss

**Fig. 5:**
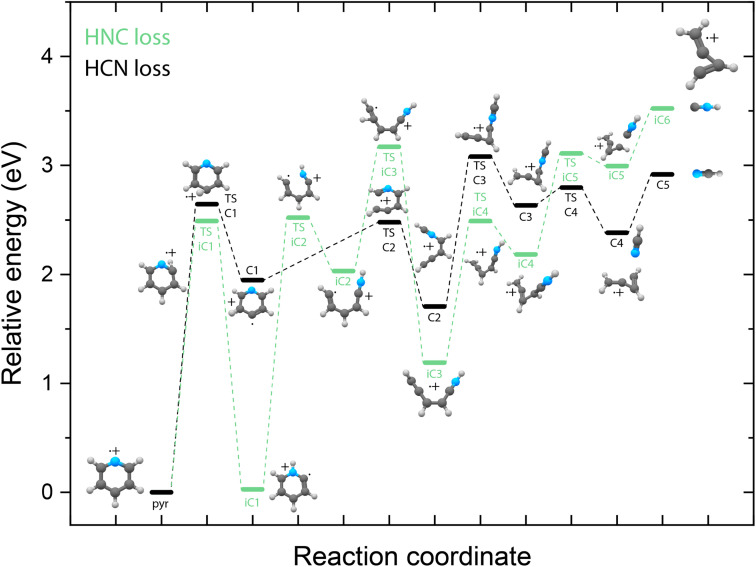
Potential energy surface for the fragmentation to methylene-cyclopropene˙^+^ from pyridine˙^+^ leading to either HCN loss (C-pathway, black pathway) or HNC loss (iC-pathway, green pathway). The energies are calculated at the B3LYP-GD3/N07D level of theory and are corrected for the zero-point vibrational energy.

**Fig. 6:**
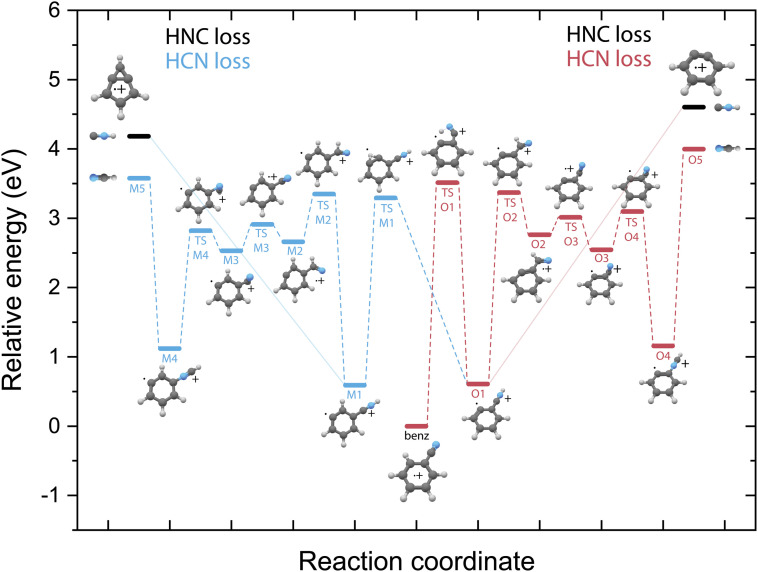
Potential energy surface for the fragmentation to both *o*-benzyne˙^+^ (red pathway) and bicyclic *m*-benzyne˙^+^ (blue pathway) from benzonitrile˙^+^ leading to either HCN loss (O-pathway, red), (M-pathway, blue) or HNC loss (black energy levels, solid lines). The energies are calculated at the B3LYP-GD3/N07D level of theory and are corrected for the zero-point vibrational energy.

Identifying the structure of the cationic fragments has revealed the formation of many cyclic and non-cyclic structures. The accompanying neutral fragment can be deduced from these structural assignments for some of the channels. However, the exact nature of the neutral counter fragment in the major loss channels leading to methylene-cyclopropene˙^+^ (from pyridine) and *o*-benzyne˙^+^/bicyclic *m*-benzyne˙^+^ (from benzonitrile) cannot be directly deduced from the spectral assignment. Both the loss of HCN and HNC could occur, each leading to different fragmentation mechanisms. To study these different pathways, we have constructed the PESs using DFT calculations, as outlined in the Supplementary Methods,† guided by the experimentally obtained structures, leading to either the HCN or HNC neutral product for both pyridine (Fig. 5) and benzonitrile fragmentation (Fig. 6).

Due to the energy difference between HCN and HNC, HCN being lower by 59 kJ mol^−1^ (0.6 eV),^76,77^ the total energy of the cationic fragment and the HNC neutral is higher compared to the HCN loss pathway. However, the potential existence of higher lying transition states on either pathway can influence the kinetics and thereby the observed fragment structures. The additional internal energy after the ionization process can be up to around 5(2) eV, which is sufficient to surmount the calculated barriers in the HCN/HNC loss PESs of pyridine and benzonitrile. Two fragmentation pathways to methylene-cyclopropene˙^+^ that lead to the two neutral isomers have been found. Both pathways start with hydrogen migration either to the nitrogen (TS iC1) or onto the ring to create a CH_2_ moiety (TS C1). This is followed by ring opening by TS C2 and TS iC2 to yield analogue structures C2 and iC3. The HCN loss pathway has been described earlier by Yim *et al.*^41^ showing a reverse barrier for formation (TS C3). On the other hand, the HNC loss pathway stays below the energy of the exit channel without any reverse barrier. Furthermore, on the HNC potential, we can locate two potential precursors, iC2 or iC3, for the H-loss product, *cis*-CHCCHCHCNH^+^, that we have observed experimentally. The relative energy of this H-loss product is calculated at 3.83 eV and can be formed upon the loss of a hydrogen atom from both the iC2 and iC3 intermediates. Similarly, using the experimental fingerprint of *m*/*z* 78, we can also exclude analogue H-loss fragments from the HCN pathway intermediate C2, iso *cis*-CHCCHCHCNH^+^ (Supplementary Fig. 7†). For the dissociative ionization of aniline, it has been observed that the H and HNC loss channels are coupled *via* the same intermediate structures.^32^ In that case, no direct H-loss was observed, but both H-migration and isomerization steps occurred prior to the loss of a hydrogen atom. Although the end products of the HNC loss pathway are energetically less favourable, the intermediates such as iC1 and iC3 are significantly lower in energy than their corresponding structures on the HCN pathway. Together with the experimental detection of the H-loss product, *cis*-HCCCHCHCNH^+^, which can exist on the same potential, it points towards the existence of the HNC pathway. This indicates that for pyridine, not the thermodynamically favored products (which is neutral HCN) are formed but the higher lying HNC isomer. However, we cannot exclude the HCN pathway as we do not have experimental evidence on the neutral structure.

Unlike on the PES for the pyridine fragmentation, the HNC loss pathways for benzonitrile do not require many isomerization steps. The fragmentation towards HNC starts with a hydrogen migration step to the nitrogen atom (TS O1), and in the case of the *m*-benzyne pathway an additional hydrogen migration on the ring (TS M1) is required, and is followed by the direct loss of HNC from the intermediates O1 or M1 leading to the *o*-benzyne˙^+^ and bicyclic *m*-benzyne˙^+^ products, respectively. These two distonic benzonitrile isomers (O1/M1) exist as reaction intermediates at relatively low energies. For protonated benzonitrile^+^, where protonation has occurred on the CN group, Wincel *et al.*^78^ has shown that upon high internal energies a similar entropically favored direct HNC loss is likely. In the case of the HCN loss pathways, for both cationic fragments the CN group needs to rotate in order to form neutral HCN. This requires multiple transition states, however all lying below the energy of the end products. The HNC loss pathway, where one isomerization step is required followed by the ejection of HNC would be a significantly faster process compared to the loss of HCN that requires more steps prior to dissociation and is expected to be slower due to steric effects, in particular the accessibility of the different atoms within the molecule that are involved in the isomerization process. The identified structures of the H-loss fragments of benzonitrile suggest a direct H-loss mechanism. In contrast to pyridine (and aniline^32^) no precursor for H-loss can therefore be observed in the PES.

### Molecular dynamics simulations of fragmentation

We have tried to simulate the observed fragmentation chemistry of pyridine and benzonitrile by running extensive MD/DFTB simulations (see Supplementary Methods†) in order to estimate the capability of such an approach to accurately predict the fragment channels and their accompanying molecular structures.

First, both the fragmentation of the canonical pyridine˙^+^ ion and its energetically low-lying alpha-distonic pyridine˙^+^ were studied. A maximum contribution of 20% of the alpha-distonic pyridine˙^+^ can be expected experimentally^79^ which can contribute differently to the fragmentation chemistry. Similar dissociation pathways with the two sets of parameters were found and the results for the alpha-distonic˙^+^ ions revealed a better agreement with the structures determined in Fig. 3. With the same energy input, the dissociation of the alpha-distonic pyridine˙^+^ ions turned out to be more efficient than that of the canonical pyridine˙^+^. The determined branching ratios (BRs) and kinetics for an internal energy of 6.6 eV are reported in Supplementary Fig. 17.†

As can be seen in Supplementary Fig. 17,† the loss of HCN/HNC as the major channel is similar to the experimental results (Fig. 1a). For the pyridine˙^+^ fragmentation, both HCN and HNC losses were observed, the majority being the loss of HCN (83% HCN *vs.* 17% HNC with Set2 at 6.6 eV and 79% HCN *vs.* 21% HNC with Set1 at 6.74 eV. Set1 and Set2 refer to two sets of SCC-DFTB parameters, see the Supplementary Methods†). Major loss of HNC was observed from the alpha-distonic pyridine˙^+^ isomer (99.8% of HNC at 500 ps). Interestingly, isomerization of pyridine˙^+^ into the alpha-distonic pyridine˙^+^ isomer was found to occur for Set1 for which the energy difference was found to be in better agreement with the DFT results (see Supplementary Methods†). Two examples of reaction pathways starting from the canonical pyridine˙^+^ from the simulations are reported in Supplementary Fig. 19.† Both pathways involve H migration and ring opening to form C_4_H_4_˙^+^ in a non-cyclic form. The cyclic form was occasionally observed in the simulations, but the non-cyclic form was found to be the final one at 1 ns. The remaining internal energy for the majority of the C_4_H_4_˙^+^ fragments was determined to be between 2–2.5 eV. This is sufficient to both induce ring-opening processes, such as calculated for methylene-cyclopropene˙^+^ (barrier of 1.8 eV)^61^ and further H migration processes (barrier of 1.8 eV) to form other linear species.

**Fig. 7:**
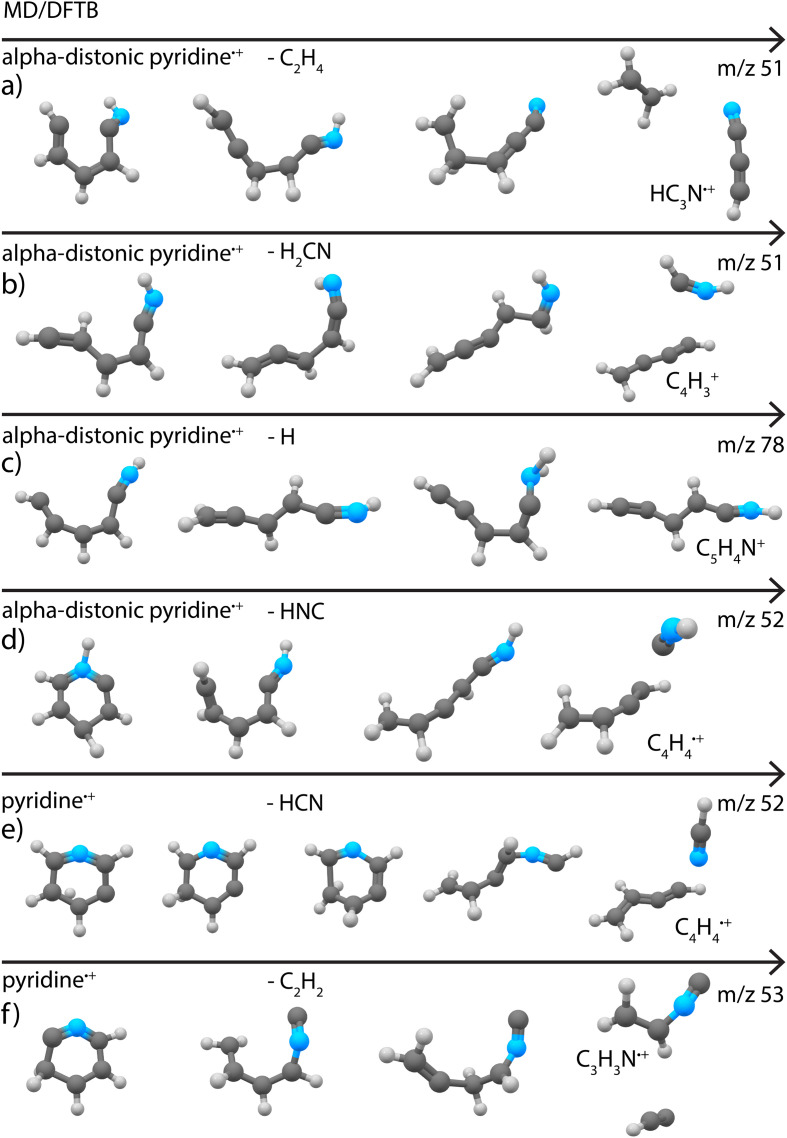
Exemplary pathways for loss of (a) C_2_H_4_, (b) H_2_CN, (c) H and (d) HNC loss from alpha distonic pyridine˙^+^ and (e) HCN and (f) C_2_H_2_ loss from canonical pyridine˙^+^ as observed in the MD/DFTB simulations at 6.74 eV internal energy of the respective cation using Set1 parameters. The arrows indicate time.

Loss of C_2_H_4_ (or H_2_CN˙ in one simulation) was found to be a minor fragment channel. Interestingly, the two products with *m*/*z* 51 reported in Fig. 3a were found to be formed from the alpha-distonic pyridine˙^+^ (Fig. 7a and b). The simulation also predicts the formation of the isocyanoacetylene˙^+^ (Supplementary Fig. 20†), whose formation cannot be completely experimentally ruled out (Supplementary Fig. 9†).

The loss of C_2_H_2_ was also observed as a minor fragment channel for both pyridine˙^+^ and the alpha-distonic pyridine˙^+^ (Fig. 7f and Supplementary Fig. 21†). The formed *m*/*z* 53 fragment from pyridine˙^+^ contains a similar CCNC backbone as the structure observed experimentally (Fig. 3c), only differing by a H migration. The fragment structure resulting from the alpha-distonic pyridine˙^+^ (Supplementary Fig. 21†) is not identified in the experiment (Supplementary Fig. 8†), making it more probable that this experimentally observed fragmentation channel goes *via* the canonical pyridine˙^+^ rather than *via* H migration and the alpha-distonic pyridine˙^+^ isomer.

The loss of H was observed as a minor channel in all simulations. Both the direct loss of H, preserving the cyclic structure of the *m*/*z* 78 cationic fragment, and the formation of non-cyclic fragments resulting from H migrations and ring opening were observed (Fig. 7c). The simulated non-cyclic structure is observed experimentally as one of the isomers (Fig. 3d).

The experimentally observed structures are more consistent with an initial C–N breaking, favored in the case of alpha-distonic pyridine˙^+^, than with an initial C–C breaking, initiated by a H migration towards the carbon atom (Supplementary Fig. 20†). Most of the fragmentation pathways proceed through the formation of the alpha-distonic pyridine˙^+^ and subsequent dissociation, as was simulated for the fragments with *m*/*z* 78, 52 and 51. Stronger spectral signatures of the alpha-distonic pyridine˙^+^ isomer have been experimentally observed at higher electron energies,^79^ which could mean that isomerization and subsequent fragmentation occurs *via* the distonic isomer at the electron energy of 50 eV where the lower abundant H, C_2_H_2_ and C_2_H_4_ loss channels have been probed.

**Fig. 8:**
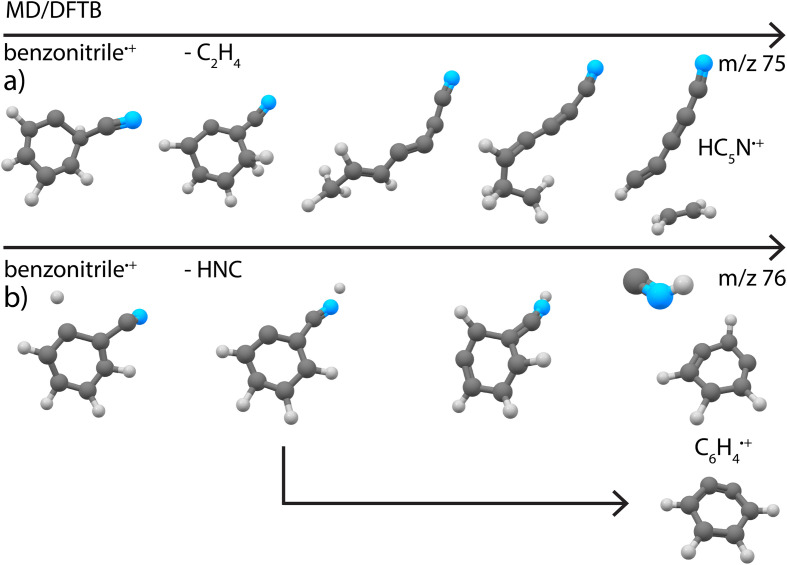
Exemplary pathways for loss of (a) C_2_H_4_ and (b) HNC from canonical benzonitrile˙^+^ as observed in the MD/DFTB simulations at 8.8 eV internal energy of the respective cation using Set1 parameters. The arrows indicate time.

Secondly, simulations of the dissociation of benzonitrile^+^ showed that the major loss channel, similar to pyridine^+^, is the loss of HCN/HCN (Fig. 8b). Both HCN and HNC losses were observed (Supplementary Fig. 22†). However, for Set1 for which the energy difference between benzonitrile and its alpha-distonic isomer is lower and more consistent with DFT results, HNC is the major product (see Supplementary Table 1†). HCN loss is major for Set2 where the formation of the alpha-distonic isomer is energetically less favorable (see Supplementary Methods†). Both the cationic *m*- and *o*-benzyne˙^+^ species were observed at the lowest energy for Set1, whereas only *o*-benzyne˙^+^ was observed for Set2 (with one non-cyclic form). When the energy increases, non-cyclic forms of C_6_H_4_˙^+^ were observed for both sets. Also, the H˙ and CN˙ direct losses were observed as minor channels (see Supplementary Table 1†) to lead to molecular ions with structures similar to those experimentally observed at *m*/*z* 102 and 77, respectively (Fig. 4d and c). Also, the loss of C_2_H_4_ was observed, leading to the cyanodiacetylene˙^+^ structure that was discovered experimentally for *m*/*z* 75 (Fig. 4a) (exemplary pathway in Supplementary Fig. 8a†).

As for the case of pyridine, the pathways are driven by H migrations that lead to C–C or C–N weakening with opening and formation of a linear chain in the case of the loss of C_2_H_4_. A few other minor channels that were only abundant in very minor quantities in the experiments were seen in the simulations (Supplementary Table 1†).

This study shows that the MD/DFTB simulations provide fragment channels leading to specific products that are in most cases also experimentally observed. Slightly alternative fragments are also observed in the MD/DFTB simulations, for example structures that encountered different degrees of H-migration processes, which may be due to some uncertainties in the DFTB potential or to differences between the experimental conditions and those of the simulations, *e.g.*, the available internal energy after electron impact ionization. The MD/DFTB simulations can give insights into dissociation mechanisms and fragment structures that could be hardly guessed intuitively due to chemical complexity. The fragment structures can be further optimized with other commonly used DFT methods and their calculated infrared spectra or rotational constants can be compared with experimental data.

### Astrophysical implications and conclusions

Although both pyridine and benzonitrile exhibit similar (partial) ionization cross sections,^80^ their formed fragments are clearly distinct (*e.g.* cyclic/non-cyclic/different *m*/*z*). Mapping the different fragmentation products of the two astrochemically relevant molecules pyridine and benzonitrile can help to understand the detection or non-detection in different astronomical environments. Similar to cyano-substituted aromatics that are proxies for their pure carbon forms, abundances of specific ‘fragment’ species in the investigated top-down processes could point towards the former existence of their parent molecules. For example, the small hydrocarbon ion l-C_3_H^+^ has been detected in the Horsehead PDR as evidence of top-down hydrocarbon chemistry, where it is expected to be a fragment from larger PAHs.^23^*Vice versa*, the knowledge of the most common fragment products can also help to construct reverse ion-molecule formation processes. For most of the HCN/HNC loss pathways that resulted in the C_4_H_4_˙^+^ and C_6_H_4_˙^+^ fragments for pyridine and benzonitrile, respectively, no reverse barriers have been found in the PESs. Only for the C_4_H_4_˙^+^ + HCN fragment channel a small barrier of 15.9 kJ mol^−1^ (0.16 eV) is present that can slow down the direct reverse formation of pyridine˙^+^ when both the cationic and neutral fragment collide reactively.

A good example is the rich chemistry in the cold molecular cloud TMC-1 where recently small hydrocarbons such as *o*-benzyne,^81^ cyclopentadiene^82^ and (cyano-substituted) PAHs^5,82,83^ have been detected. This source was already known for the presence of many large neutral cyanopolyyne molecules such as HC_3_N, also isocyanoacetylene HC_2_NC (ref. 84) and HC_5_N, with an odd number of carbons up to the largest found chain HC_11_N (ref. 85) and also some of their protonated forms, HC_3_NH^+^ and HC_5_NH^+^ (tentatively).^86^ It is speculated that larger (linear) chains can undergo cyclization, which is thermodynamically favorable.^87^ It is also discussed that the fragmentation products of larger cyclic species can lead to modest-sized molecules that can be important precursors for small aromatic molecules, but are not (accurately) considered in the models.^88^ The interplay between these two bottom-up and top-down viewpoints is still not clear. From this study we can conclude a few points that can support this discussion.

Firstly, we can identify two cationic cyanopolyyne species, HC_3_N˙^+^ and HC_5_N˙^+^, as fragmentation products of pyridine and benzonitrile, respectively. When starting from species with more carbons, such as cyano-substituted PAHs, we can expect even larger cyanopolyyne species as fragments.

Secondly, the most abundant fragment from the dissociative ionization of benzonitrile is the *o*-benzyne˙^+^ radical cation. Interestingly, the neutral form has recently been detected in TMC-1.^81^ Besides neutral-radical bottom-up formation reactions, *o*-benzyne could also be formed from dissociative electron recombination of phenyl^+^,^81^ which we here identified as a fragment of benzonitrile. This (cationic) *o*-benzyne structure is not only an important fragment observed here, but also an essential precursor for PAH growth. This has already been demonstrated experimentally where the PAH indene was formed upon the reaction of *o*-benzyne and the allyl radical.^89^ The cationic form formed here from benzonitrile can be regarded as even more reactive, either with H_2_, possibly forming benzene, or with other small neutral hydrocarbons to form PAHs. In addition, for the H-loss channel of benzonitrile we observe no ring-opening but direct H removal which maintains the cyclic structure of the parent. Altogether, we form different fragments, *o*-benzyne˙^+^, phenyl^+^ and dehydro-benzonitrile^+^ which are all likely to react with H_2_ and/or CN˙ radicals and/or undergo (dissociative) electron recombination to form molecules such as benzene^90^ and sequentially benzonitrile.^91^

For pyridine, the H-loss and C_2_H_2_ loss fragments are non-cyclic species which are less likely to directly react back to the cyclic parent. They can react with H_2_ to form more stable and more saturated carbon or nitrile species but, on the other hand, play a role as reactive intermediates in other bottom-up growth processes. One interesting example shown in this study is the fragmentation product with *m*/*z* 53 from pyridine, assigned to two protonated isocyanide acetylene^+^ isomers. Interestingly, fragmentation studies of pyrimidine also showed the formation of these isomers containing the CCNC backbone.^92^ Experimental and theoretical studies on the C_3_H_3_N^+^ PES and ion–molecule reactions of these intermediates have shown that structures with this backbone can be important intermediates for reactions with HCN to form the diazine species pyrazine˙^+^ and pyrimidine˙^+^.^64,93^ All these results combined show that fragmentation of pyridine can lead to potential higher energetic intermediate species that can further react to form species with multiple incorporated nitrogen atoms, thereby leading to even more complexity.

Thirdly, the same interplay between formation and destruction is important for even smaller species such as HCN and HNC. These are among the most abundant species in interstellar space but also with significantly varying relative abundances in different environments. The HNC/HCN ratio can, *e.g.*, differ between ∼0.013 in star-forming regions^94^ and 0.54–4.5 in dark clouds.^95^ There are multiple competitive processes present and it is important, as Loison *et al.*^96^ mentioned, to look for new sources and sinks of HCN and HNC in order to understand the different isomeric ratios. Destruction mechanisms are important in the dissociative electron recombination of HCNH^+^ leading to both HCN and HNC.^97^ Also, the different photo-dissociation rates of HNC and HCN can alter the ratio when UV interstellar radiation is present.^98^ Moreover, the observationally derived abundances can be influenced by the different collisional excitation cross sections.^99^ Analogous to the speculated H_2_ formation from the photo-destruction of PAHs in PDRs,^36,100^ also the HCN/HNC fragmentation pathway of nitrogen-containing (polycyclic) aromatic molecules could play an important role in those environments. Observations have shown that the HNC/HCN ratio decreases with a factor ∼5 towards the star.^101^ Also, the CN˙ abundance increases towards the star, whereas the abundances of HCN and HNC decrease.

We can speculate that also (small) nitrogen containing molecules such as the ones discussed in this study, pyridine and benzonitrile, can add to the formation of both HCN and HNC. For both precursors we have observed that the dominant fragmentation pathways lead to the loss of HCN/HNC, demonstrating the vulnerability of the nitrogen containing aromatic molecules. This can also been seen if one compares the loss of H as the major channel in the fragmentation of benzene which has a higher appearance energy (AE = 13.7 eV)^102^ compared to the loss of HCN/HNC for pyridine (AE = 12.34 eV).^58^ Two different fragmentation pathways releasing either HCN or HNC as the neutral can be found for both molecules. If the neutral does not gain sufficient internal energy after the fragmentation, no further isomerization towards the thermodynamically stable HCN species is expected due to the significant barrier of 1.91 eV (184 kJ mol^−1^).^76^ Based on the experimentally determined structures, calculated PESs and the results of the MD/DFTB simulations, we tend to form the higher lying HNC isomer upon the fragmentation process. Also, when using a higher electron impact ionization energy of 50 eV, benzonitrile is fragmented to phenyl^+^ and CN˙ is formed, a process that can also be initiated by UV radiation from stars although the appearance energy of 13.6 eV is close to the H atom ionization energy. But to completely map the abundances of all the molecules correctly, one needs an astrochemical model that includes both bottom-up reactions and top-down processes. This study provides the structural information and branching ratios of the important top-down processes that can be used to construct such a model.

Overall, the experimental approach described here allows identification of the different fragmentation products by measuring their fingerprint infrared spectra to deduce the molecular structures. But in these spectra, more information is present, as they can be used as reference data for astronomical detections. The fingerprint infrared spectra allow to distinguish many different spectral features that are indicative for characteristic groups within the molecules. For example, the CC triple bond stretches of the cyanopolyyne˙^+^ structures are strong and informative features to look for in high-resolution astronomical infrared spectra such as those coming from JWST. Also, many of the discovered fragments possess a sufficient permanent dipole moment required for radio-astronomical observations. A particular interesting species is the most abundant fragment of pyridine, the cyclic methylene-cyclopropene˙^+^. This molecule is stabilized due to the aromatic character of the three-membered cyclopropenylium moiety.^61^ In the atmosphere of Titan, a possible precursor of this species, c-C_3_H_3_^+^, cyclopropenyl^+^, has been detected by *in situ* mass-spectrometry.^103^ Possible derivatives of this molecule such as methyl-cyclopropenyl^+^ (C_4_H_5_^+^) or the here identified methylene-cyclopropene˙^+^ (C_4_H_4_˙^+^) are discussed to exist as well. Methylene-cyclopropene˙^+^ in Titan’s atmosphere can also be an important fragmentation proxy for the presence of pyridine, as protonated pyridine has also been found there using mass-spectrometry.^104^

**Fig. 9:**
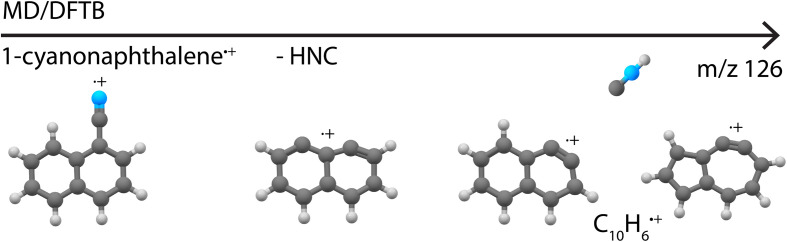
Cationic fragment structures upon the loss of HNC from canonical 1-cyanonaphthalene˙^+^ as observed in the MD/DFTB simulations at 12.7 eV internal energy of the respective cation using Set1 parameters.

Besides providing infrared reference spectra, and to structurally assign molecules formed in the dissociative ionization, this study also allowed us to benchmark MD-DFTB modeling to predict the structures of the fragments. The good agreement between the outcome of the simulations and the spectroscopic assignments encourages us to propose dissociation pathways for larger and more complex species such as the recently astronomically detected PAH 1-cyanonaphthalene.^5^ Exploratory simulations for the dissociation of 1-cyanonaphthlene˙^+^ (C_11_H_7_N˙^+^) were performed for internal energies of 12.7 eV and 13.6 eV with Set1 parameters (720 simulations of 1 ns were run). At both energies, the major loss channel of HNC/HCN was observed with for instance 105 C_10_H_6_˙^+^ fragments at 1 ns at 12.7 eV, where HNC was found to be the major neutral loss channel with 100% HNC and no HCN at 12.7 eV, and 94% HNC and 6% HCN at 13.6 eV. At the higher energy of 13.6 eV, the loss of H, CN˙ and C_2_H_2_ appeared (Supplementary Table 2†). The structures of the potentially important cationic fragments of the HNC loss channel are shown in Fig. 9 (exemplary pathways in Supplementary Fig. 23†), showing both didehydronaphthalene (arynes) cation structures, as well as the formation of isomers containing five- and seven-membered rings. These MD/DFTB simulations provide a valuable basis to experimentally study the dissociative ionization of cyanonaphthalenes with the same multi-faceted approach as presented here for the monocyclic pyridine and benzonitrile species, and to investigate if similar structural motifs as observed here can be found in the dissociation of larger N-heterocyclic and cyano-substituted polycyclic aromatic hydrocarbons.

## Author contributions

D. B. R.: conception and design of work, data collection, data analysis and interpretation, theoretical calculations, visualization, drafting and editing of article. A. S.: theoretical calculations, data interpretation, editing of article. K. S.: theoretical calculations, data interpretation, editing of article. J. G. M. S.: data collection, data analysis and interpretation, editing of article. B. R.: funding acquisition, project administration, supervision, data interpretation, editing of article. S. B.: conception and design of work, data collection, data interpretation, methodology, project administration, funding acquisition, supervision, drafting and editing of article. All authors discussed the manuscript.

## Conflicts of interest

The authors declare no competing interests.

## Supplementary Material

FD-245-D3FD00015J-s001
