# Supplementary material for: Fingerprinting fragments of fragile interstellar molecules: dissociation chemistry of pyridine and benzonitrile revealed by infrared spectroscopy and theory
Source: Faraday Discuss. 2023 Mar 22;245:221–44. doi: 10.1039/d3fd00015j (PMC10510038; doi:10.1039/d3fd00015j)
Supplement: FD-245-D3FD00015J-s001 [file FD-245-D3FD00015J-s001.pdf]

Electronic Supplementary Material (ESI) for Faraday Discussions.
This journal is © The Royal Society of Chemistry 2023

Supplementary Information:

Fingerprinting fragments of fragile interstellar molecules: dissociation chemistry of pyridine and benzonitrile revealed by infrared spectroscopy and theory

Daniël B. Rap[1], Aude Simon[2], Kim Steenbakkers[1], Johanna G.M. Schrauwen[1], Britta Redlich[1] and Sandra Brünken[1]*

[1]Institute for Molecules and Materials, FELIX Laboratory, Radboud University, Toernooiveld 7, 6525 ED Nijmegen, The Netherlands, *E-mail: sandra.bruenken@ru.nl

[2]Laboratoire de Chimie et Physique Quantiques (LCPQ), Fédération FeRMI, CNRS & Université Toulouse III – Paul Sabatier, 118 Route de Narbonne, 31062 Toulouse, France

Supplementary Methods

Spectroscopic measurements

Experiments were performed in a cryogenic 22-pole ion trap instrument coupled to the FELIX free-electron lasers described in more detail earlier[1]. Vapors of pyridine and benzonitrile were led into the ion source where the molecules were fragmented using dissociative electron impact ionization with 15 or 50 eV electrons. Under the experimental conditions, we expect a mixture of 80 % canonical and a maximum of 20 % of the alpha-distonic isomer for pyridine[2,3] The benzonitrile cation is determined here to be purely the canonical structure (Supplementary Fig. 2). For most of the fragment masses, a direct electron impact ionization source was used. For the m/z 51, 53 and 78 fragments from the dissociative ionization of pyridine, a Gerlich-type electron impact storage ion source was used[4]. The comparability of the two sources was assessed by measuring the infrared spectrum of m/z 52 from pyridine formed in both sources (Supplementary Fig. 4). From these measurements, similar abundances for the main isomer were measured. More details are shown in Supplementary Fig. 5 and 6. The fragments of interest were mass-selected by a quadrupole mass spectrometer and led into the cryogenic 22-pole ion trap[53]. The ions were trapped and cooled down to around 6-7 K by a pulse of He:Ne (3:1 ratio). The formed ion-Ne complexes were studied using IRPD spectroscopy using the free-electron laser at the FELIX Laboratory[5]. The ions were irradiated with intense (up to 30 mJ) and tunable infrared radiation provided by FEL-2 operating at 10 Hz in the 550-2200 cm^{-1} range with a typical 0.5% FWHM bandwidth. When the laser is resonant with a vibrational mode of the ion-Ne complex, the amount of depletion is measured as a function of the wavelength to yield an infrared spectrum. The wavelength is calibrated using a grating spectrum analyser and the ion signal is normalized to the laser pulse energy (E), and number of FELIX pulses (N) to determine the relative intensity (I) according to:

$$\mathrm{I} = -\frac{\ln\left({}^{\mathrm{S}}/_{\mathrm{B}}\right)}{\mathrm{E}*\mathrm{N}},$$

with S the observed ion counts and B the baseline ion count number. To increase the match with theoretical calculations, some of the spectra have been also calibrated for the photon flux by multiplying the normalized intensity (I) with the photon energy ($h\nu$), were ν is the frequency of the light in wavenumbers.

A saturation depletion measurement[6] has been performed to measure the abundancies of the two isomers in the m/z 52 channel. Multiple laser pulses, resonant with a vibrational mode of one isomer, were used to fully deplete this active isomer. The analysis of this depletion as a function of the deposited energy yielded the abundance of the active isomer (Supplementary Fig. 5 and 6).

Quantum chemical calculations

Structures of relevant cations, inspired from literature, where optimized to their lowest energy structure using density functional theory (DFT) using Gaussian 16[7].

For most of the molecules the functional/basis set combination B3LYP-GD3/N07D was used to perform the geometry optimization and the vibrational frequency calculations as it showed to be a robust method for various cationic molecules[8–11]. A typical scaling factor of 0.976 was used to account for anharmonicity. For some of the ions anharmonic treatment of the vibrational modes, using the VPT2 functionality of Gaussian 16, showed a significant improvement and is shown as the calculated spectrum. For the non-linear closed-shell species CH_2CCCH^+ with m/z 51, the B2PLYPD3/aug-cc-pVTZ functional and basis set combination was used[12].

The Renner-Teller and spin-orbit splitting patterns of the bending modes of $HC_3N^{\bullet+}$ ($^2\Pi$) and $HC_2NC^{\bullet+}$ ($^2\Pi$) with m/z=51 and $HC_5N^{\bullet+}$ ($^2\Pi$) with m/z 75 were calculated with an effective Hamiltonian approach similar to that of Steenbakkers et al.[13] For this work no cross-mode Renner-Teller term was assumed so that the splitting of each of the bending modes could be calculated separately. The Hamiltonian for mode k then reduces to:

$$H_{eff} = H_{vib,k} + H_{SO} + H_{RT,k},$$

where $H_{vib,k}$ is the vibrational Hamiltonian, H_{SO} the spin-orbit Hamiltonian and $H_{RT,k}$ the Renner-Teller Hamiltonian. The expressions are given below:

$$H_{vib,k} = \omega_k(v_k + 1)$$

$$H_{SO} = A_{SO}\hat{L}_z\hat{S}_z$$

$$H_{RT,k} = \frac{1}{4}\varepsilon_k\omega_k(L_-^2 q_{+,k}^2 + L_+^2 q_{-,k}^2)$$

Here the nomenclature of Steenbakkers et al.[13] was followed. The spectroscopic parameters ε_k, ω_k and A_{SO}, represent the Renner Teller constant, the harmonic vibrational frequency and the spin-orbit constant, respectively. For $HC_5N^{•+}$ all spectroscopic constants were taken from Gans et al.[14] For $HC_3N^{•+}$ and $HC_2NC^{•+}$ the harmonic vibrational frequencies and their corresponding Renner-Teller constants were determined based on frequency calculations on the RCCSD(T)-F12a/cc-pVTZ-F12 level of theory. The spin-orbit constant used for $HC_3N^{•+}$ was taken from Steenbakkers et al.[13] and was assumed to be equal for $HC_2NC^{•+}$. The intensity ratio of the bending modes of all three species was calculated at the B2PLYPD3/aug-cc-pVTZ level of theory.

The minima structures and transition states for the PESs were calculated at the B3LYP-GD3/N07D level of theory and the corresponding minima were connected using intrinsic reaction coordinate (IRC) calculations. All energies have been corrected for the zero-point vibrational energy.

Molecular dynamic simulations

In order to explore the observed dissociation pathways and kinetics, extensive on-the-fly Born Oppenheimer molecular dynamics (MD) simulations with the electronic structure computed at the SCC-DFTB level of theory - hereafter quoted MD/DFTB - were run using the deMonNano code.[15] We showed that using the original mio set of parameters[16] led to discrepancies with experiments regarding the H loss vs. the C_2H_2 loss for the dissociation of PAHs[17], leading us to scale the C-H atomic integrals by a scaling factor (0.95) in order to decrease the strength of the C-H bond with respect to that of the C-C bond. Using such a Hamiltonian led to better agreement with experimental results[18,19]

Similarly, in this work, we consistently adjusted the parameters for the N-H bond as explained in the next paragraph.

Two sets of parameters, Set1 and Set2, were used. The default atomic integral values $\langle \varphi_\mu^X | \hat{h}_0 | \varphi_\mu^H \rangle$ and $\langle \varphi_\mu^X | \varphi_\mu^H \rangle$, where φ_μ^X are the atomic orbitals of X=C,N,H and $\hat{h}_0$ the monoelectronic Hamiltonian, were scaled by 0.95 for X=C,N,H in the Set2 parameters set, consistently with our previous work[19]**.** However, the energy difference between cationic benzonitrile and the corresponding alpha-distonic ion was found too high compared to DFT results (84 kJ/mol vs. 49 kJ/mol[20]). In order to recover the DFT energy difference, $\langle \varphi_\mu^X | \hat{h}_0 | \varphi_\mu^H \rangle$ and $\langle \varphi_\mu^X | \varphi_\mu^H \rangle$ were scaled by 0.95 for X=C,H and 0.98 for X=N. Besides, charge model3 (CM3) charges were used instead of the original Mulliken charges in the SCC-DFTB Hamiltonian to improve the description of the polarity of the bonds as in previous works ($q_{cm3}=q_{Mull} + d_{XY}$ with $d_{NH}=0.120$[21]**,** $d_{CH}=0.09$[22], and $dN_C=0.08$). With this final set of parameters named hereafter Set1, the energy difference between cationic benzonitrile and the corresponding alpha-distonic ion was computed to be 41 kJ/mol. Similarly, Set1 parameters provide a better description of the energy difference between cationic pyridine and the corresponding alpha-distonic ion (+4.4 kJ/mol) whereas with Set2, an energy difference of 44 kJ/mol is obtained, our reference being the DFT value (with ZPE corrections) of -5.8 kJ/mol[2]. However, with both sets of parameters HNC becomes too low in energy compared to HCN (with Set2: δE (HNC-HCN) = +9.9 kJ/mol while with Set1 δE (HNC-HCN) = -56 kJ/mol), the reference value being +62 kJ/mol[23]. This illustrates the limits of transferability of the SCC-DFTB parameters when different types of bonds are involved. However, for these systems accurate energetics of the minima and the corresponding barriers of the entire ion are more important in order to simulate the fragmentation correctly.

Several hundreds to 1800 simulations of 300 ps to 1 ns with initial random atomic velocities were ran in the microcanonical (NVE) ensemble for several internal energies. The SCC-DFTB energy and its gradient were computed every 0.1 fs. The lowest internal energy for which fragmentation was observed for cationic benzonitrile in these timescales was determined to be 8.0 eV. Several energy values were investigated up to 8.8 eV. The dissociation of cationic pyridine was also investigated using

360 up to 1800 MD runs using the two sets of parameters for the same energies per bond as in the case of the lowest energy simulations of cationic benzonitrile, corresponding to internal energies of 6.60 to 6.74 eV for cationic pyridine.

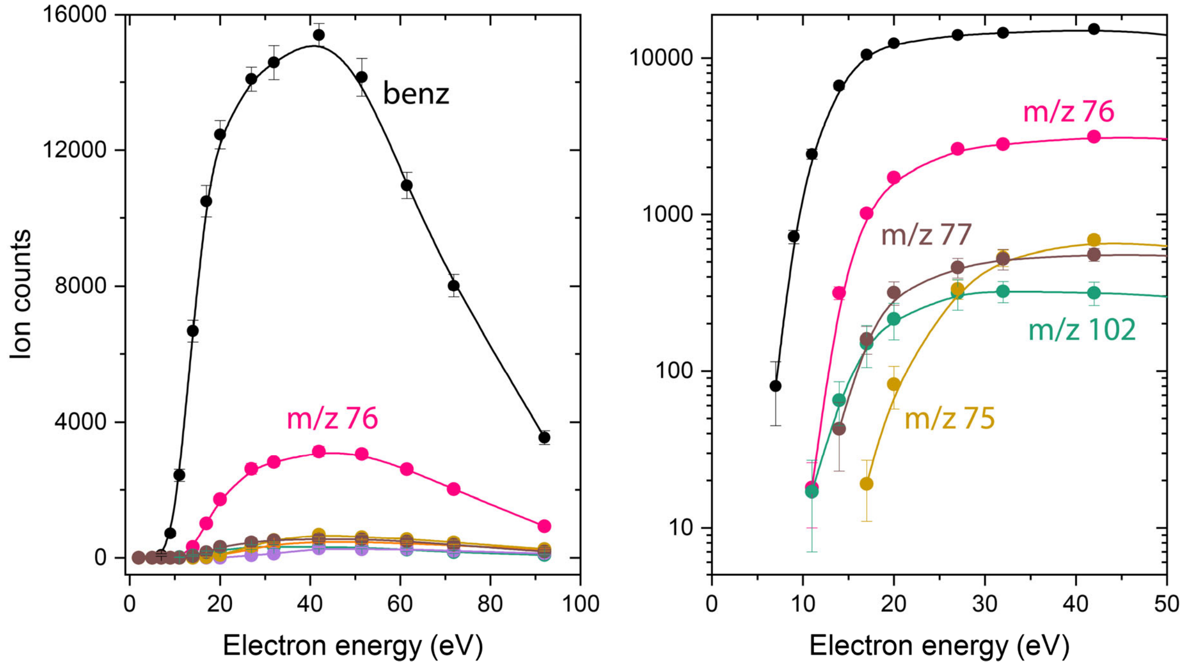

Supplementary Fig. 1: Experimental breakdown curves of the dissociative ionization of benzonitrile. The right panel shows an inset of the 0-50 eV region. An experimental uncertainty of 2 eV is expected for this electron impact ionization source.

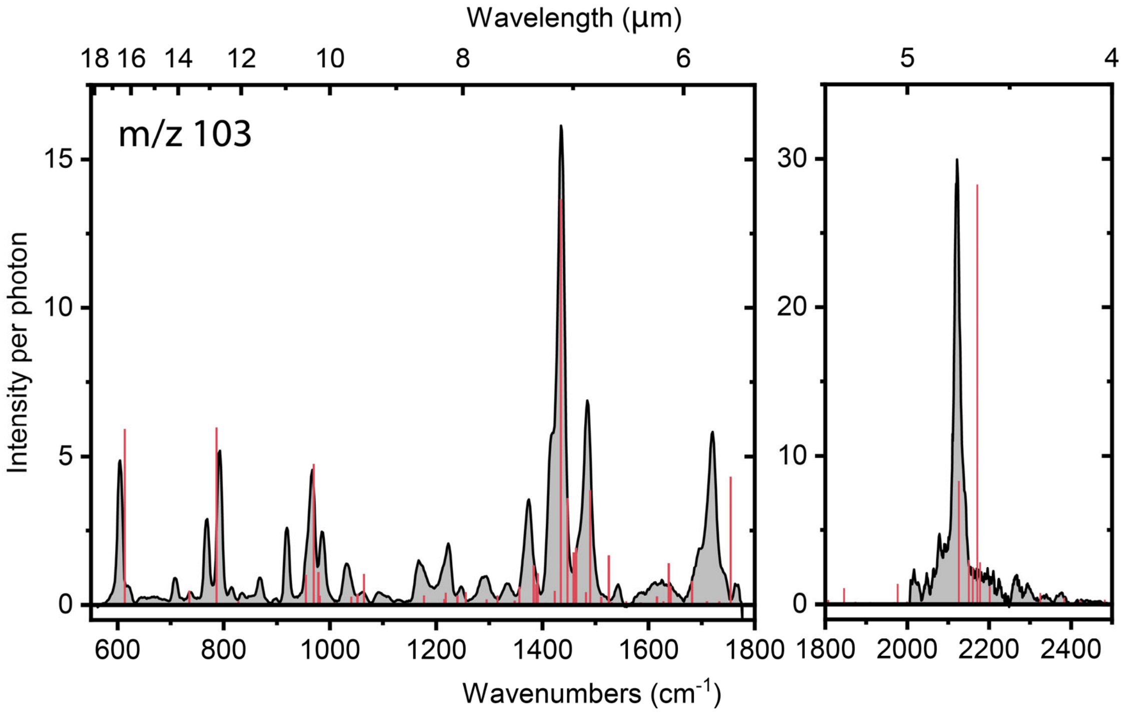

Supplementary Fig. 2: Experimental infrared spectrum of benzonitrile$^{\bullet+}$ (grey). Comparison with anharmonic calculated vibrational frequencies at the B3LYP-GD3/N07D level of theory (red). We can assign the spectrum to the canonical benzonitrile radical cation without the presence of distonic isomers. A more detailed spectroscopic analysis will be presented in a forthcoming paper.

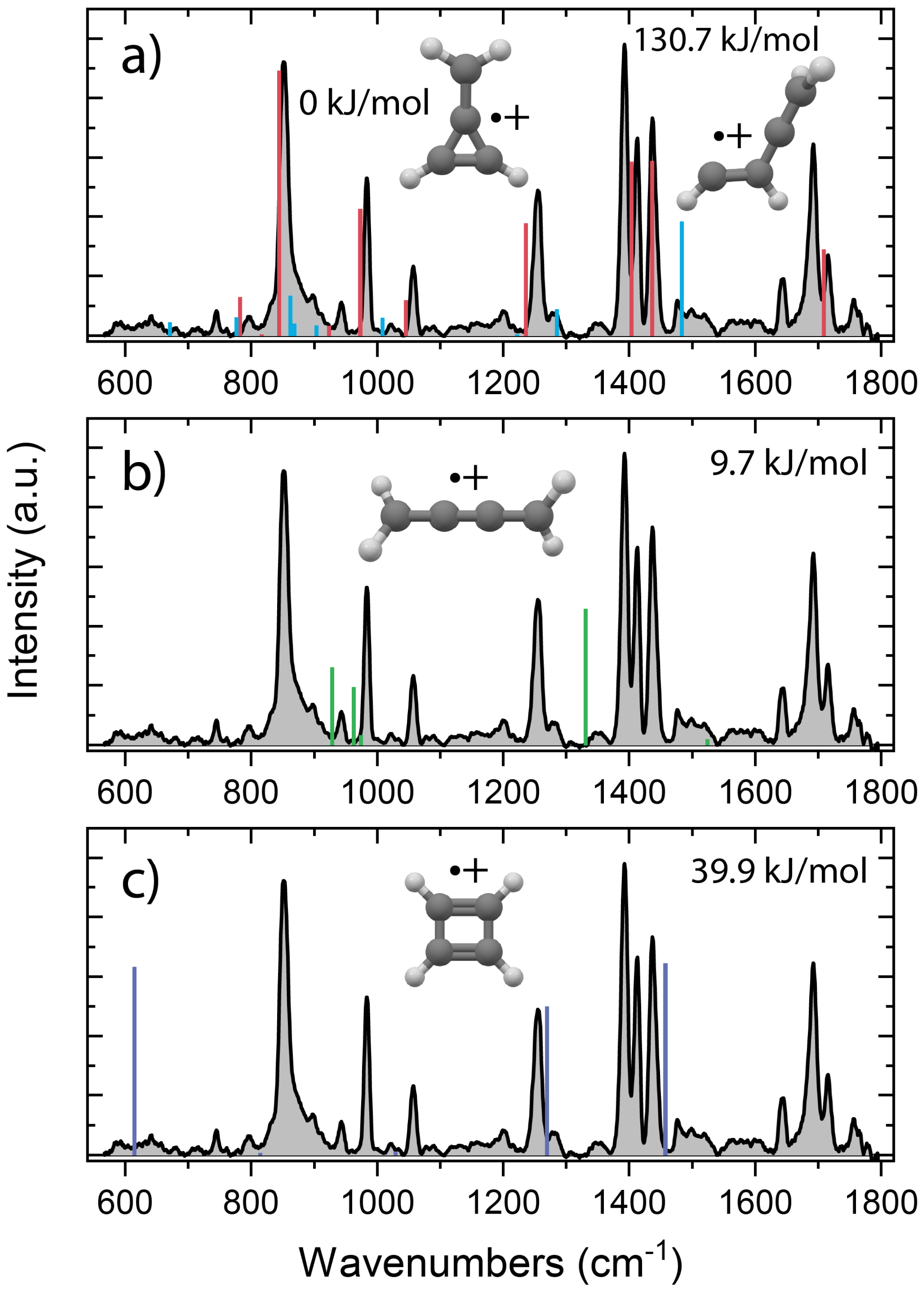

Supplementary Fig. 3: Experimental infrared fingerprint spectrum (grey) of the **m/z 52** fragment channel of the dissociative ionization of pyridine. Calculated vibrational modes are shown for (a) the assigned methylene-cyclopropene$^{\bullet+}$ (c-$C_3H_2(CH_2)^{\bullet+}$) (red), $CHCHCCH_2^{\bullet+}$ (blue), (b) butatriene$^{\bullet+}$ ($H_2C_4H_2^{\bullet+}$) (green) and (c) cyclobutadiene$^{\bullet+}$ (c-$C_4H_4^{\bullet+}$) (purple). The infrared frequencies have been calculated at the harmonic B3LYP-GD3/N07D level of theory. The relative electronic energies for the $C_4H_4^{\bullet+}$ isomers are displayed and have been corrected for the zero-point vibrational energy.

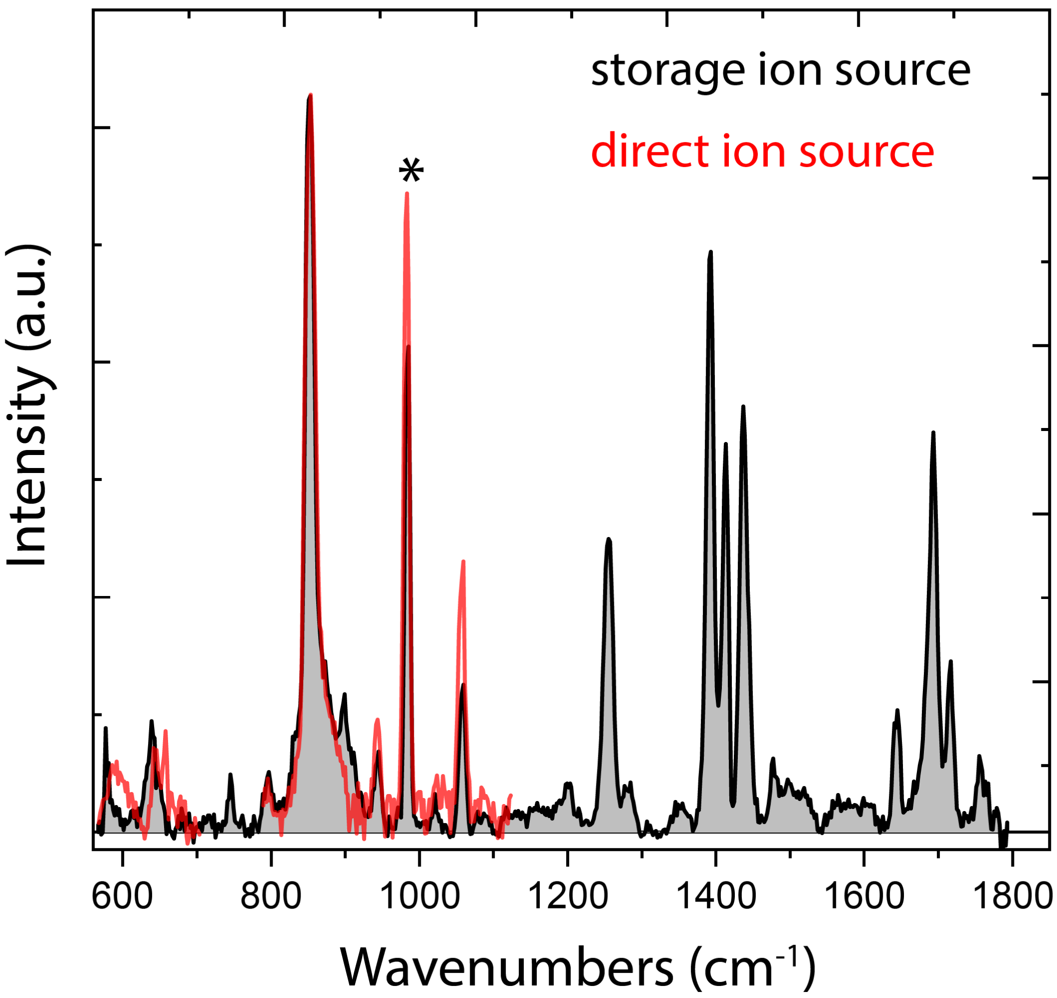

Supplementary Fig. 4: Experimental infrared fingerprint spectrum (grey) of the **m/z 52** fragment channel of the dissociative ionization of pyridine formed in the storage ion source with 15 eV electrons. Comparison with the experimental infrared fingerprint spectrum (red) of the **m/z 52** fragment formed in the direct ion source using 15 eV electrons. The individual spectra have been normalized for the power and the photon flux. The spectrum from the direct ion source has been scaled to equal intensity of the main band around 853 cm^{-1}. The band notated with the asterisk is used to perform a saturated spectral scan as discussing in Supplementary Fig. 5.

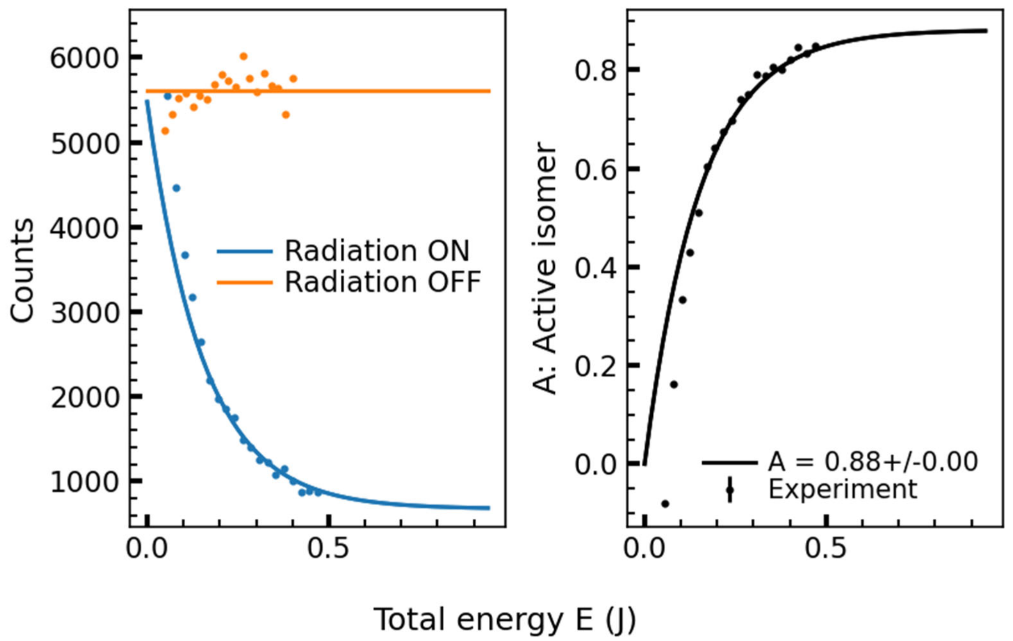

Supplementary Fig. 5: Experimental saturation depletion scan of the 1253 cm^{-1} mode of methylene-cyclopropene$^{\bullet+}$ (**m/z 52)** formed in the electron impact ion storage source with 15 eV electrons. The on-resonance scan at 1253 cm^{-1} (blue) and off-resonance scan at 1310 cm^{-1} (yellow) are shown in the left panel. The relative depletion of the active isomer (A) is shown in the right panel as a function of the deposited energy (E). The relative depletion of this band belonging to the methylene-cyclopropene$^{\bullet+}$ isomer shows saturation around 85-90 %. A saturated infrared scan across another isomer specific mode at 984 cm^{-1} (denoted by the asterisk in Supplementary Fig. 4) using the direct electron impact source showed a similar depletion value of 82 % indicating no significant differences for the fragmentation products between the two used sources.

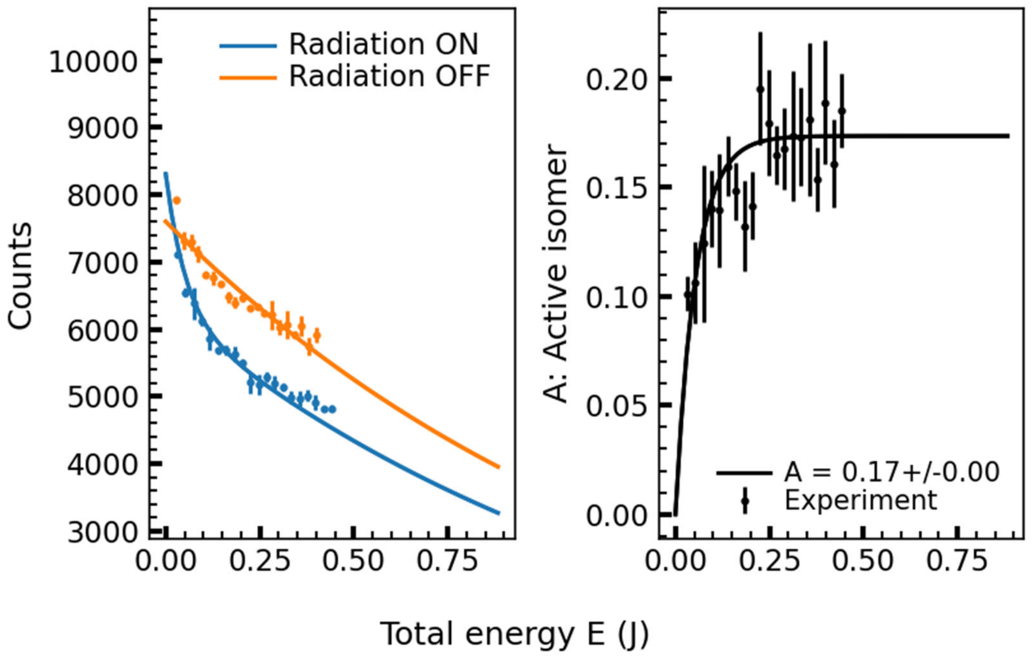

Supplementary Fig. 6: Experimental saturation depletion scan on the 1280 cm^{-1} mode of $CHCHCCH_2^{\bullet+}$ (**m/z 52)** formed in the ion storage source. The on-resonance scan at 1280 cm^{-1} (blue) and off-resonance scan at 1310 cm^{-1} (yellow) are shown in the left panel. The relative depletion of the active isomer (A) is shown in the right panel as a function of the deposited energy (E). The relative depletion of this band belonging to the $CHCHCCH_2^{\bullet+}$ isomer shows saturation around 15-20 %.

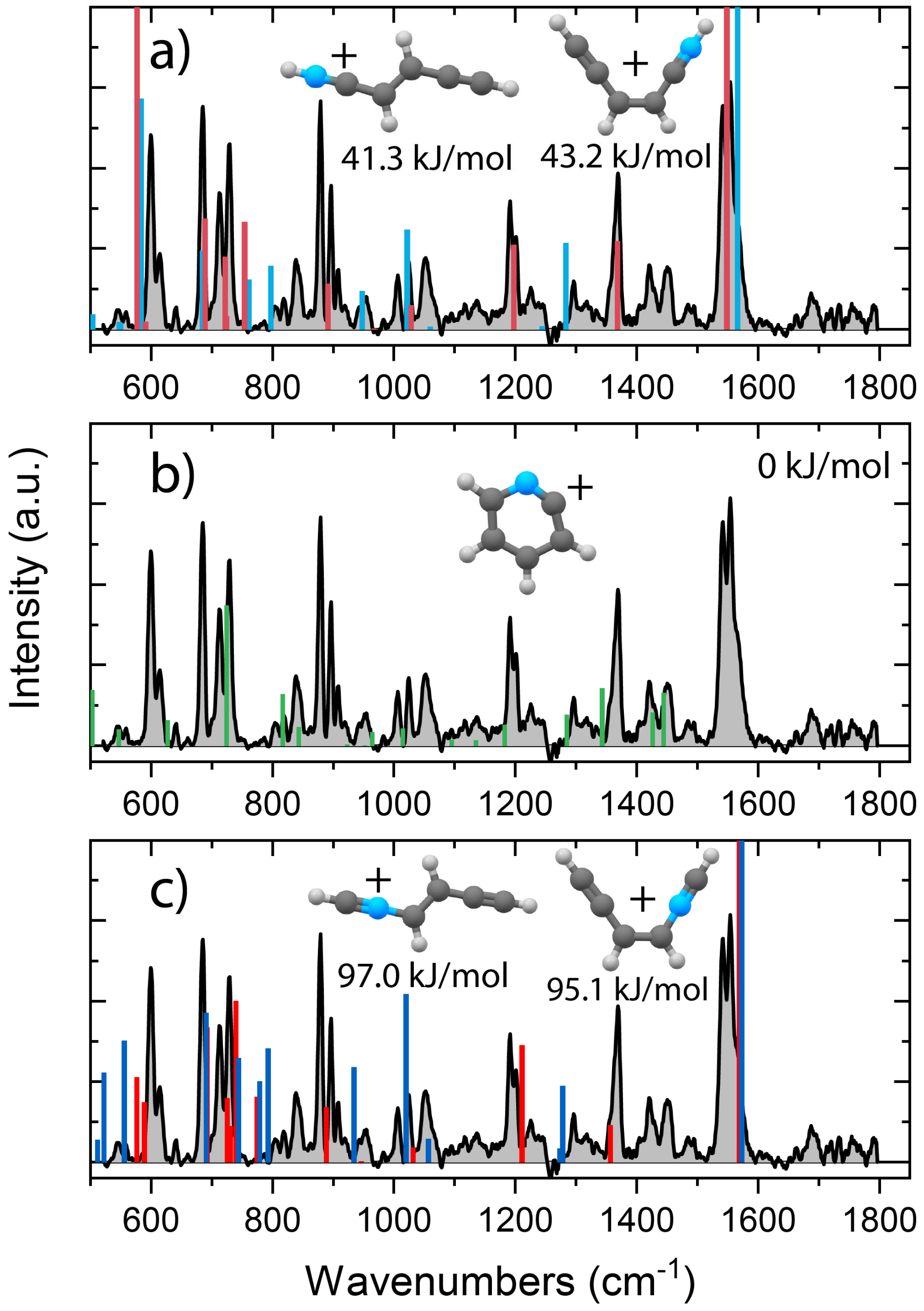

Supplementary Fig. 7: Experimental infrared fingerprint spectrum (grey) of the **m/z 78** fragment channel of the dissociative ionization of pyridine. Calculated vibrational modes are shown for (a) cis-$CHCCHCHCNH^+$ (red), trans-$CHCCHCHCNH^+$ (blue), (b) 1-dehydro-pyridine$^+$ (c-$C_5H_4N^+$) (green) and (c) iso cis-$CHCCHCHCNH^+$ (red), iso trans-$CHCCHCHCNH^+$ (blue). The infrared frequencies have been calculated at the harmonic B3LYP-GD3/N07D level of theory. The relative electronic energies for the $C_5H_4N^+$ isomers are displayed and have been corrected for the zero-point vibrational energy.

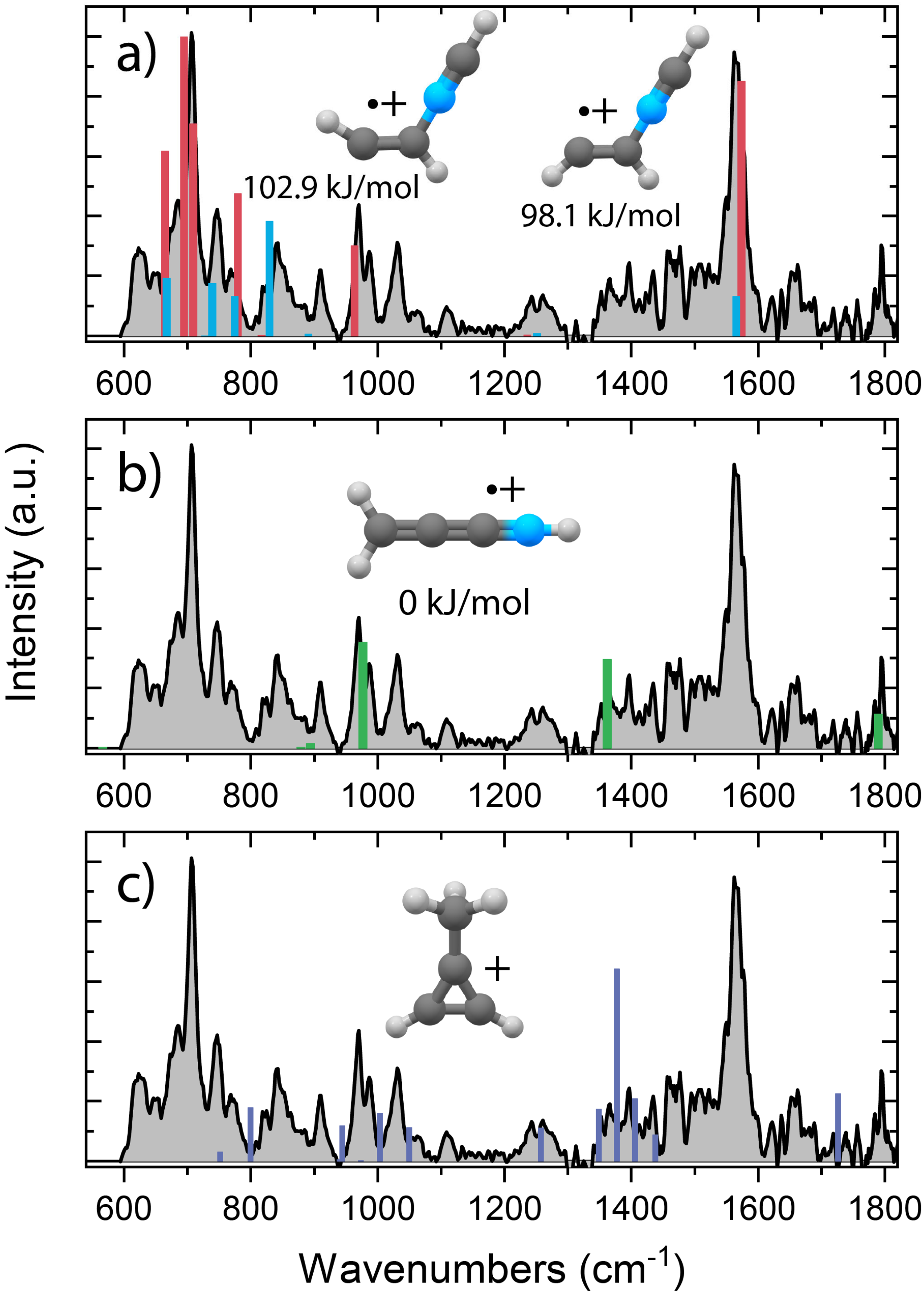

Supplementary Fig. 8: Experimental infrared fingerprint spectrum (grey) of the **m/z 53** fragment channel of the dissociative ionization of pyridine. Calculated vibrational modes are shown for (a) protonated trans-isocyanoacetylene$^+$ (trans-CHCHNCH$^+$, red), protonated cis-isocyanoacetylene$^+$ (cis-CHCHNCH$^+$, blue), (b) propadiene imine$^{\bullet+}$ ($H_2C_3NH^{\bullet+}$) (green) and (c) methyl-cyclopropene (c-$C_3H_2(CH_3)^+$) (purple). The infrared frequencies have been calculated at the harmonic B3LYP-GD3/N07D level of theory. The relative electronic energies for the $C_3H_3N^{\bullet+}$ isomers are displayed and have been corrected for the zero-point vibrational energy. The displayed $C_4H_5^+$ structure is calculated to be one of the lowest energy structures for this chemical formula.

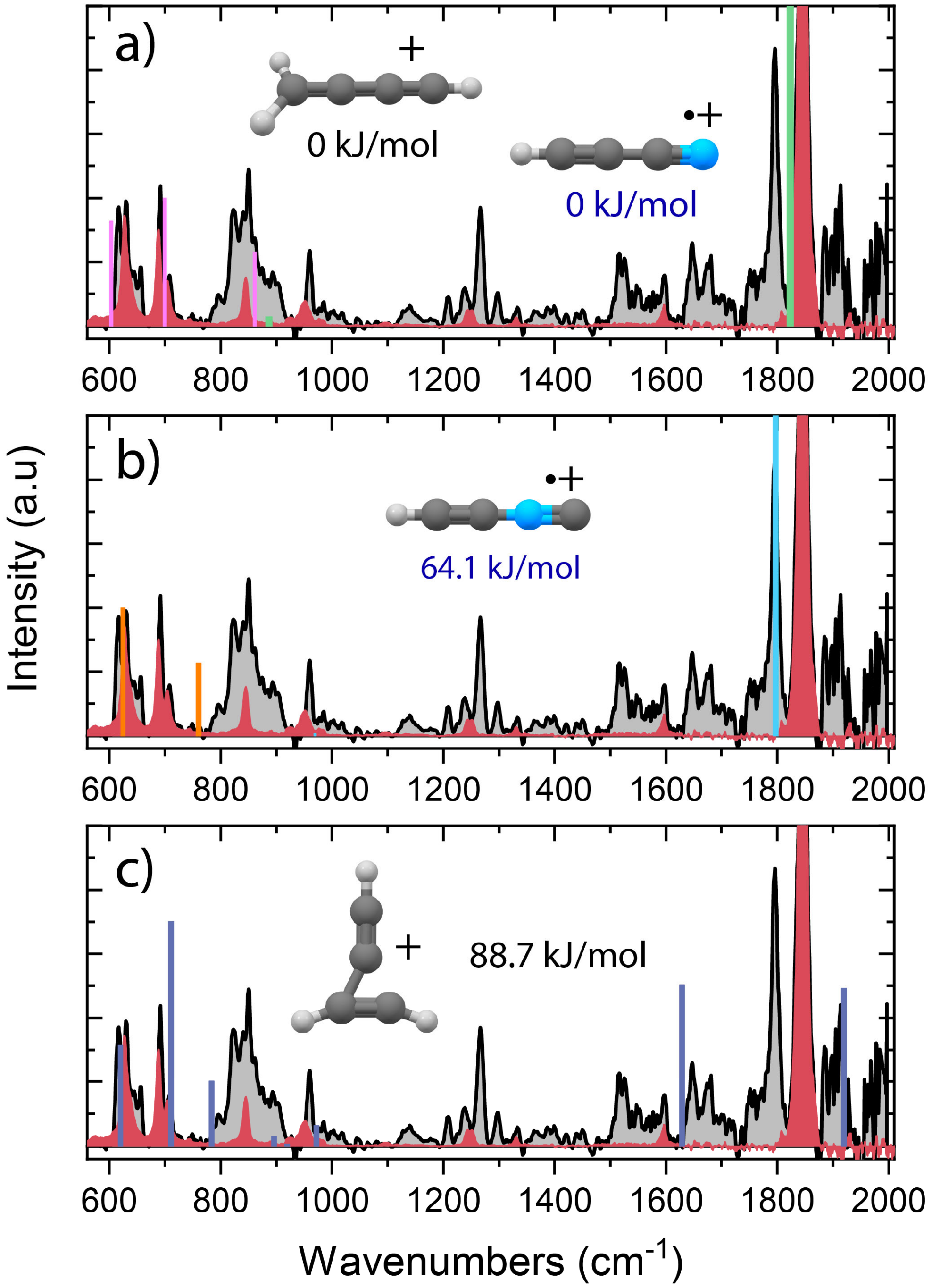

Supplementary Fig. 9: Experimental infrared fingerprint spectrum (grey) of the **m/z 51** fragment channel of the dissociative ionization of pyridine. An experimental reference spectrum of the similarly assigned cyano-acetylene$^{•+}$ ($HC_3N^{•+}$) (red trace) from Steenbakkers et al.[13] is shown in panel a. Calculated vibrational modes are shown for (a) cyano-acetylene$^{•+}$ ($HC_3N^{•+}$) (pink/green) (b) isocyano acetylene$^{•+}$ ($(HC_2NC)^{•+}$) (orange/blue) and (c) methylidyne-cyclopropene^{+} ($c\text{-}C_3H_2(CH)^{+}$) (purple). The infrared frequencies of methylidyne-cyclopropene^{+} have been calculated at the harmonic B3LYP-GD3/N07D level of theory. The Renner-Teller affected bending modes of $HC_3N^{•+}$ (pink, panel a) and $HC_2NC^{•+}$ (orange, panel b) have been determined using an effective Hamiltonian approach and are combined with harmonic stretching modes calculated at the RCCSD(T)-F12a/cc-pVTZ-F12 level of theory (green, panel a / blue, panel b, respectively. The relative electronic energies for the $C_4H_3^{+}$ isomers are displayed and have been corrected for the zero-point vibrational energy. Similarly, the relative electronic energies for the $C_3HN^{•+}$ isomers are displayed in dark blue.

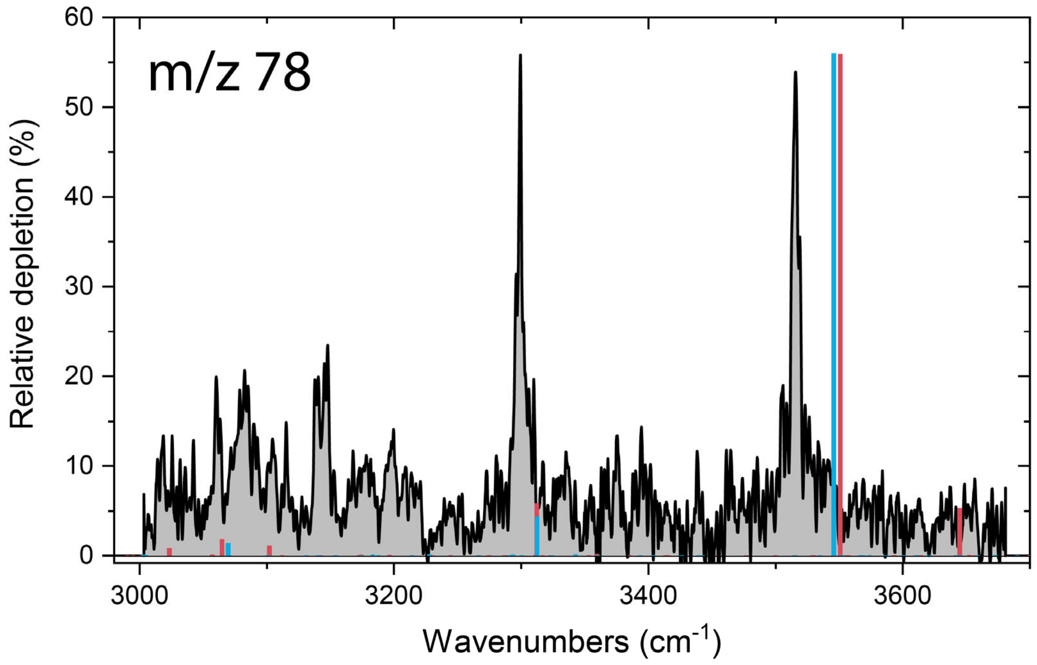

Supplementary Fig. 10: Experimental infrared spectrum (grey) in the NH stretch region of the **m/z 78** fragment channel of the dissociative ionization of pyridine. Calculated vibrational modes are shown for cis-$CHCCHCHCNH^+$ (red) and trans-$CHCCHCHCNH^+$ (blue). The infrared frequencies have been calculated at the anharmonic B3LYP-GD3/N07D level of theory.

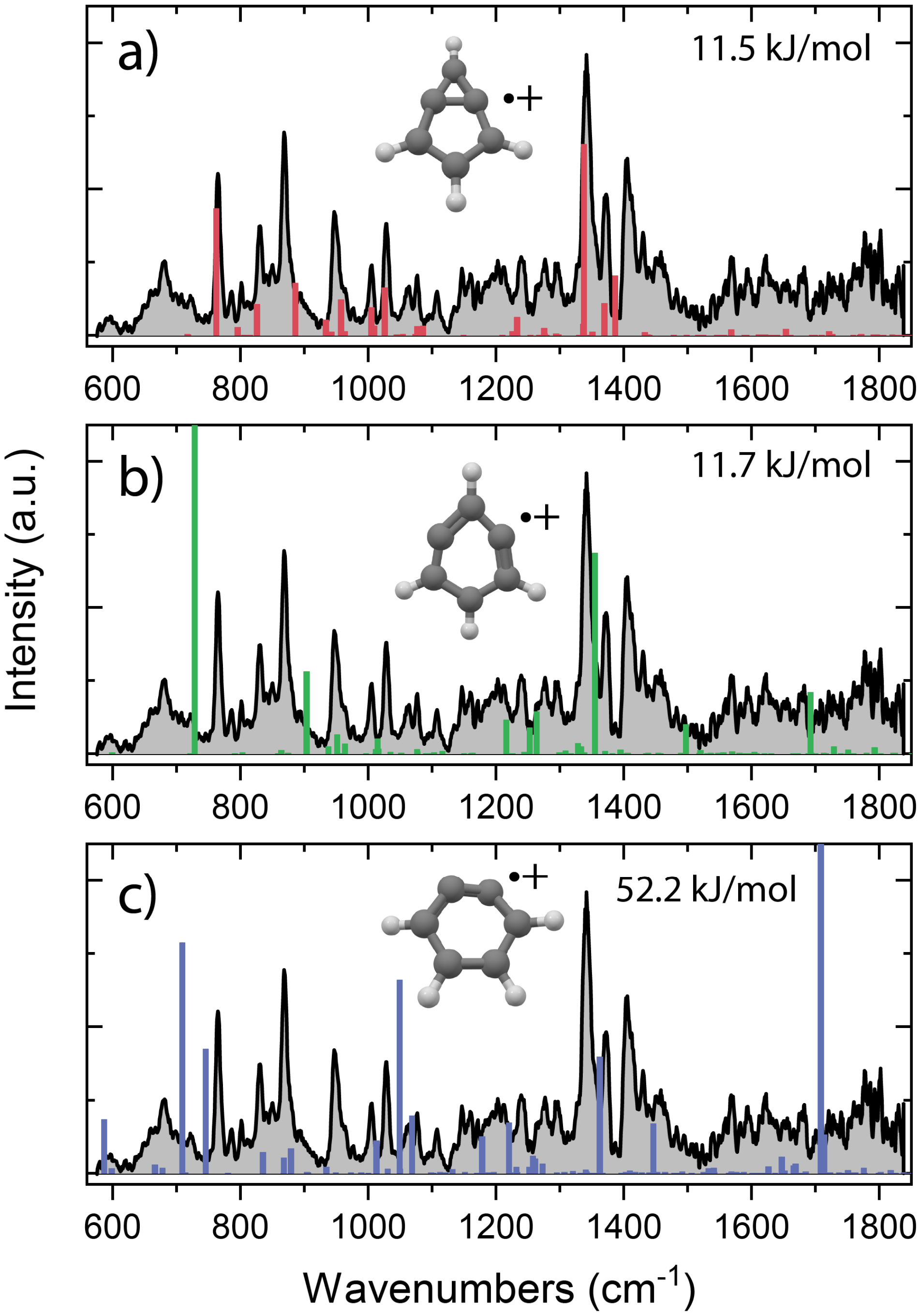

Supplementary Fig. 11: Experimental infrared fingerprint spectrum (grey) of the **m/z 76** fragment channel of the dissociative ionization of benzene. Calculated vibrational modes are shown for (a) the assigned bicyclic-m-benzyne$^{•+}$ (red), (b) m-benzyne$^{•+}$ (green) and (c) o-benzyne$^{•+}$ (purple). The infrared frequencies have been calculated at the anharmonic B3LYP-GD3/N07D level of theory. The relative electronic energies for the $C_6H_4^{•+}$ isomers are displayed and have been corrected for the zero-point vibrational energy.

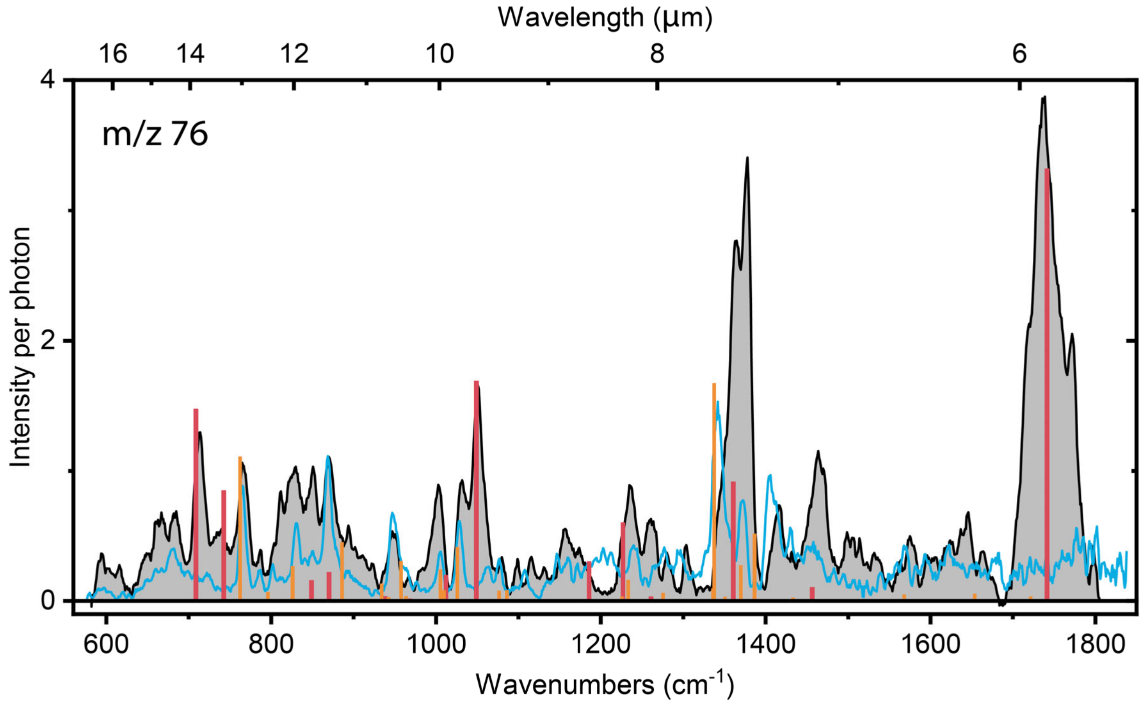

Supplementary Fig. 12: Experimental infrared fingerprint spectrum (grey) of the **m/z 76** fragment channel of the dissociative ionization of benzonitrile. Calculated vibrational modes are shown for the assigned o-benzyne$^{•+}$ (red, harmonic) and bicyclic m-benzyne$^{•+}$ (orange, anharmonic). Comparison with the m/z 76 fragment spectrum of benzene (blue) is shown. The infrared frequencies have been calculated at the harmonic B3LYP-GD3/N07D level of theory.

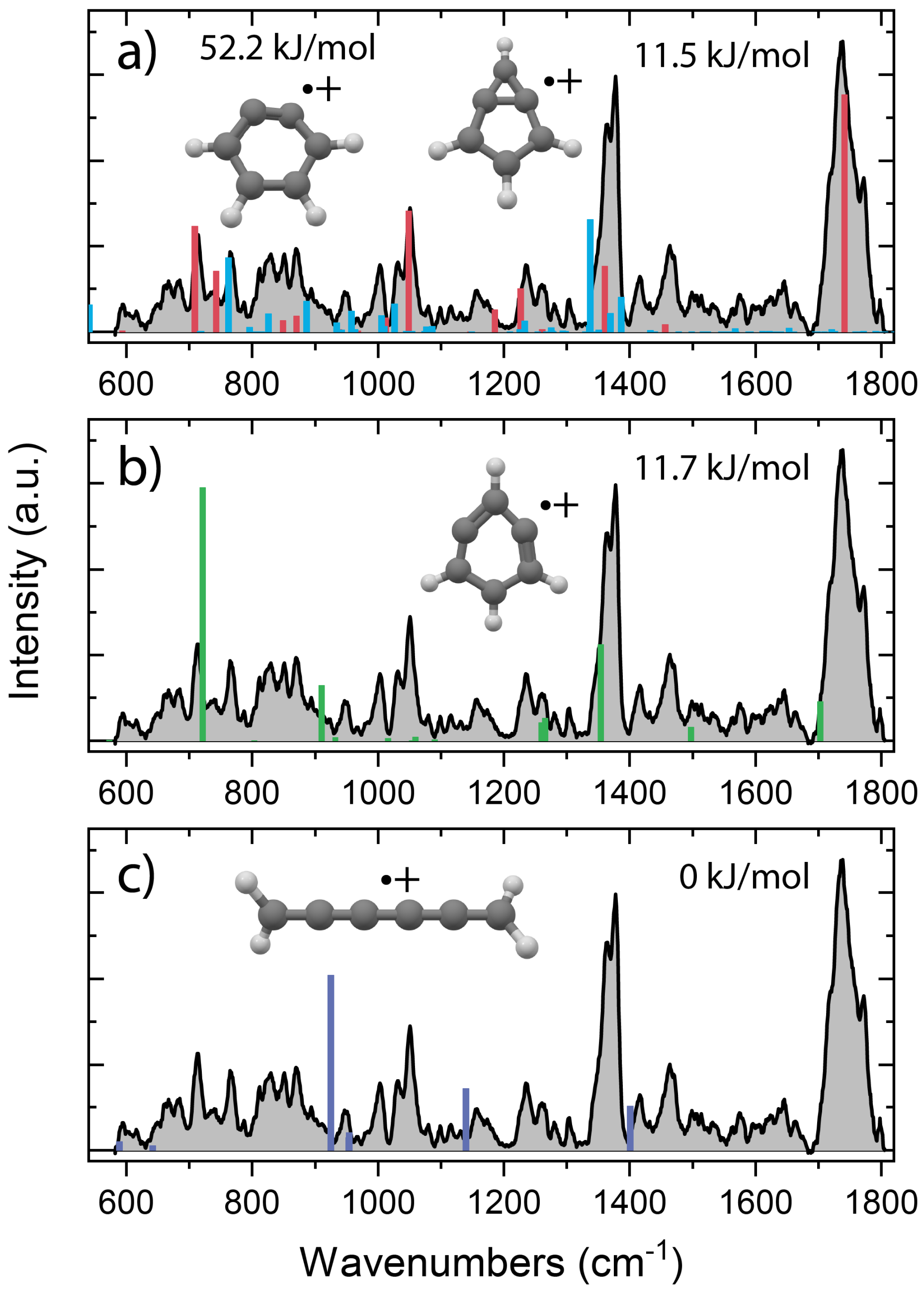

Supplementary Fig. 13: Experimental infrared fingerprint spectrum (grey) of the **m/z 76** fragment channel of the dissociative ionization of benzonitrile. Calculated vibrational modes are shown for the assigned (a) o-benzyne$^{\bullet+}$ (red) and bicyclic-m-benzyne$^{\bullet+}$ (blue), (b) m-benzyne$^{\bullet+}$ (green) and (c) hexapentaene$^{\bullet+}$ ($H_2C_6H_2^{\bullet+}$) (purple). The infrared frequencies have been calculated at the harmonic B3LYP-GD3/N07D level of theory. The vibrational modes of the bicyclic-m-benzyne$^{\bullet+}$ have been calculated using the anharmonic B3LYP-GD3/N07D level of theory. The relative electronic energies for the $C_6H_4^{\bullet+}$ isomers are displayed and have been corrected for the zero-point vibrational energy.

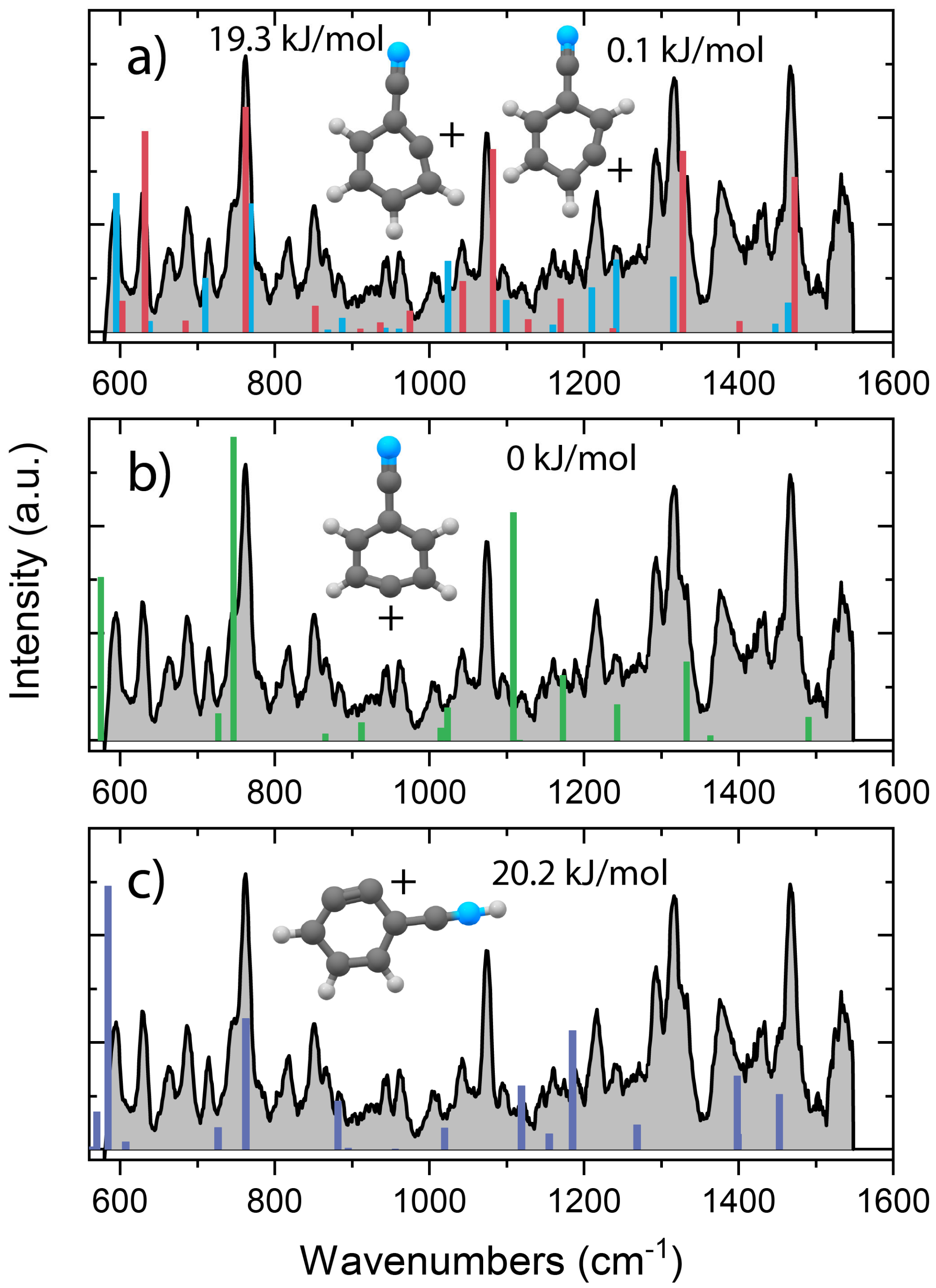

Supplementary Fig. 14: Experimental infrared fingerprint spectrum (grey) of the **m/z 102** fragment channel of the dissociative ionization of benzonitrile. Calculated vibrational modes are shown for the assigned (a) 2-dehydro (blue) and 3-dehydro-benzonitrile$^+$ (red), (b) 4-dehydro-benzonitrile$^+$ (green) and (c) 2,3-didehydro-H-benzonitrile$^+$ (purple). The infrared frequencies have been calculated at the harmonic B3LYP-GD3/N07D level of theory. The relative electronic energies for the $C_7H_4N^+$ isomers are displayed and have been corrected for the zero-point vibrational energy.

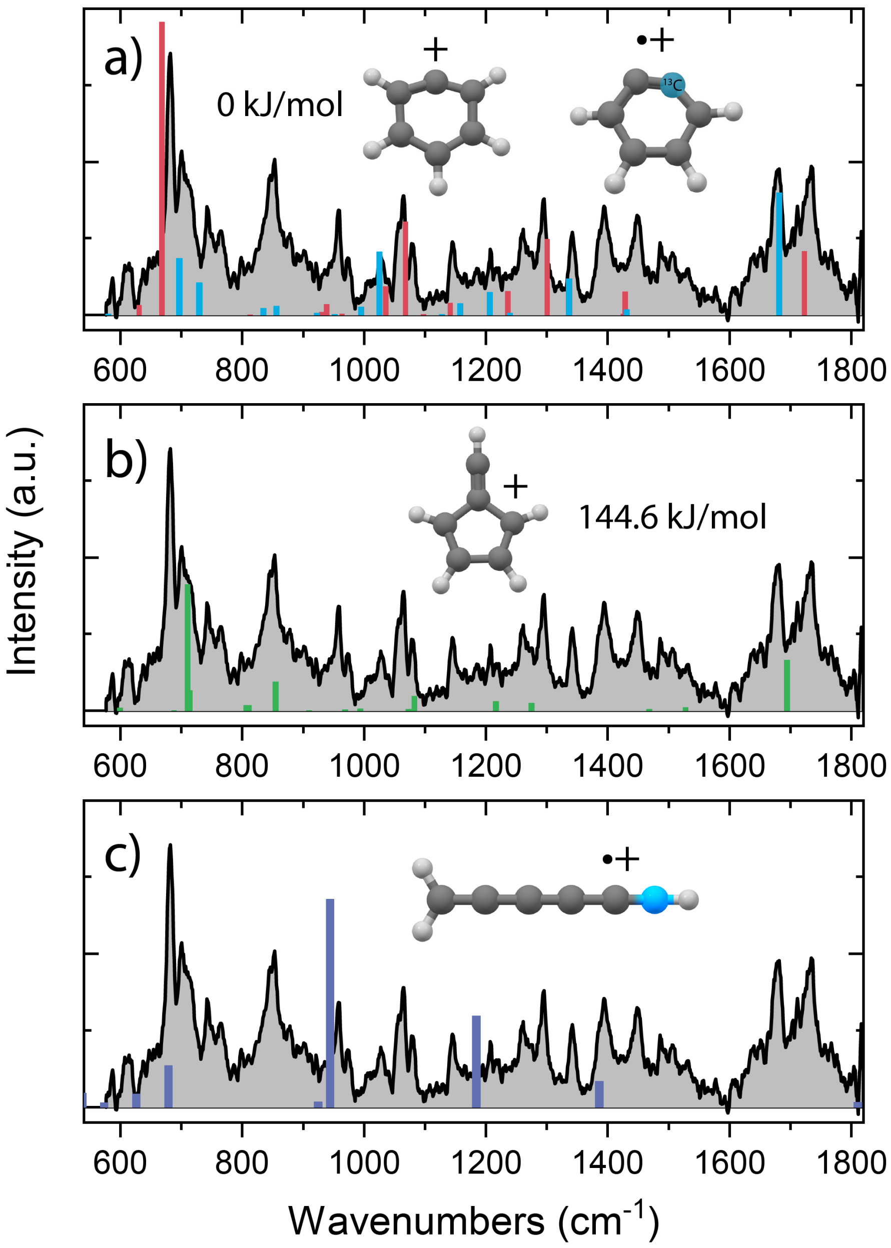

Supplementary Fig. 15: Experimental infrared fingerprint spectrum (grey) of the **m/z 77** fragment channel of the dissociative ionization of benzonitrile. Calculated vibrational modes are shown for the assigned (a) phenyl$^+$ (red) and ^{13}C-o-benzyne$^{•+}$ (blue), (b) methylidyne-cyclopentadienylium$^+$ (green) and (c) pentatetraene imine$^{•+}$ ($H_2C_5NH^{•+}$) (purple). The infrared frequencies have been calculated at the harmonic B3LYP-GD3/N07D level of theory. The relative electronic energies for the $C_6H_5^+$ isomers are displayed and have been corrected for the zero-point vibrational energy. The displayed $C_5H_3N^{•+}$ structure is calculated to be one of the lowest energy structures for this chemical formula.

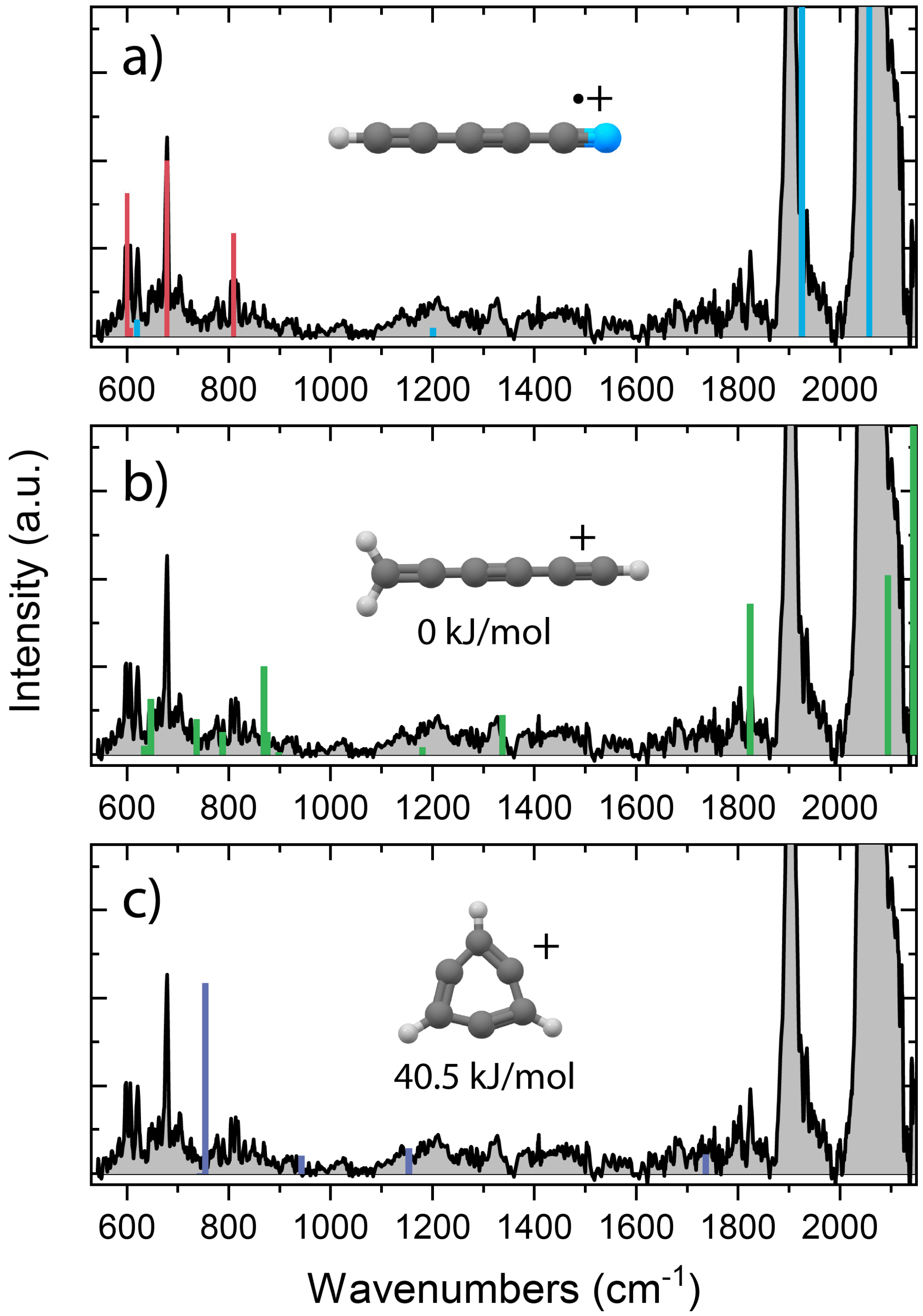

Supplementary Fig. 16: Experimental infrared fingerprint spectrum (grey) of the **m/z 75** fragment channel of the dissociative ionization of benzonitrile. Calculated vibrational modes are shown for (a) the assigned cyano-diacetylene$^{\bullet+}$ ($HC_5N^{\bullet+}$) (red/blue), (b) protonated triacetylene^{+} ($H_2C_6H^+$) (green) and (c) 3,5-di-dehydro-phenyl^{+} (c-$C_6H_3^+$) (purple). The infrared frequencies have been calculated at the harmonic B3LYP-GD3/N07D level of theory. The Renner-Teller affected bending modes of $HC_5N^{\bullet+}$ (red, panel a) have been determined using an effective Hamiltonian approach and are combined with harmonic stretching modes (blue) by Gans et al.[14]. The relative electronic energies for the $C_6H_3^+$ isomers are displayed and have been corrected for the zero-point vibrational energy. The displayed $C_5HN^{\bullet+}$ structure is calculated to be one of the lowest energy structures for this chemical formula.

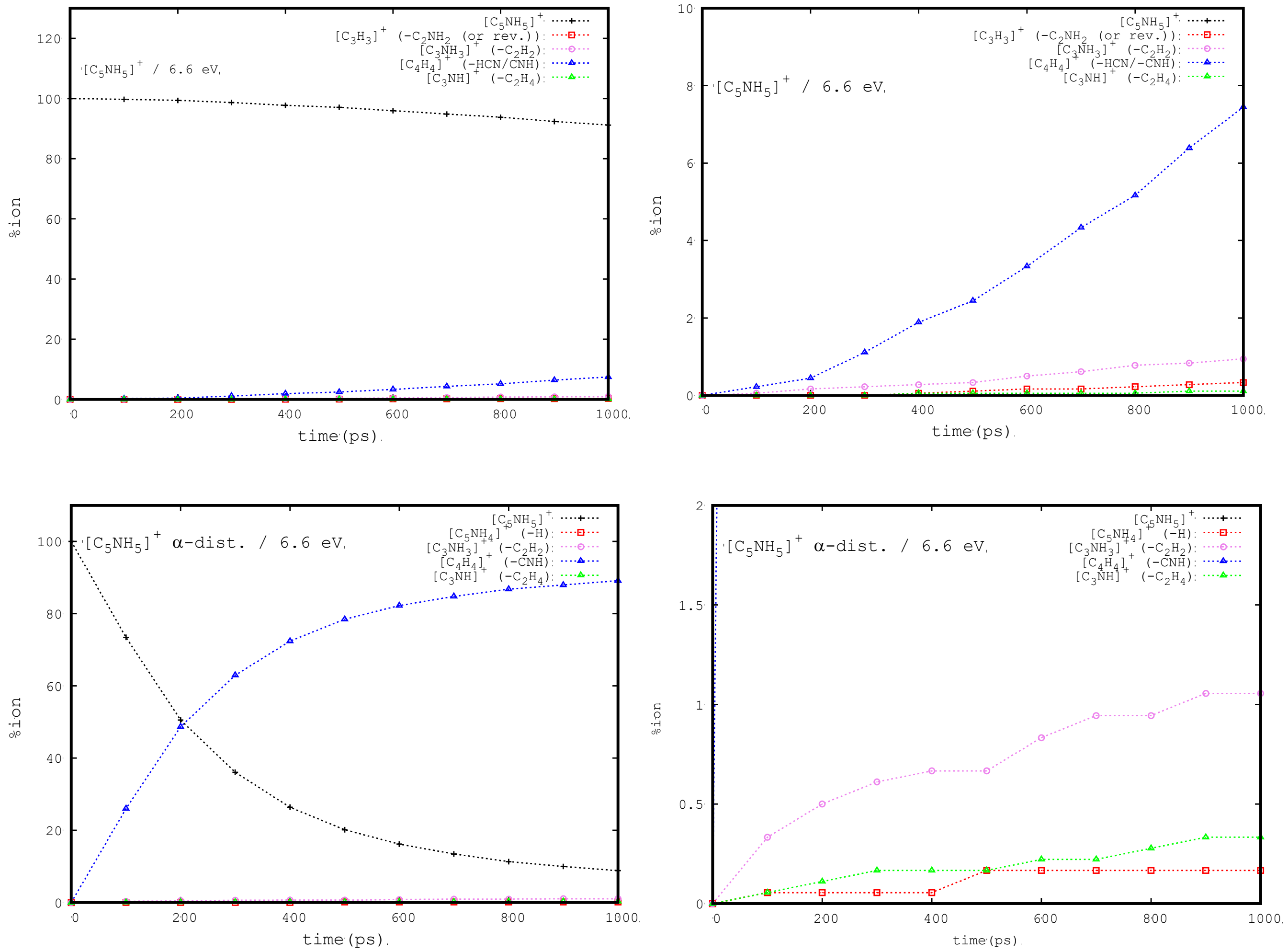

Supplementary Fig. 17: BRs and kinetics for the dissociation of the canonical pyridine$^{\bullet+}$ (top panels) compared to alpha-distonic pyridine$^{\bullet+}$ (bottom panels). 1800 simulations were performed at 6.6 eV internal energy of the respective cation for each species using Set2 parameters. Similar kinetics were observed for Set1 parameters (see Supplementary Fig. 18).

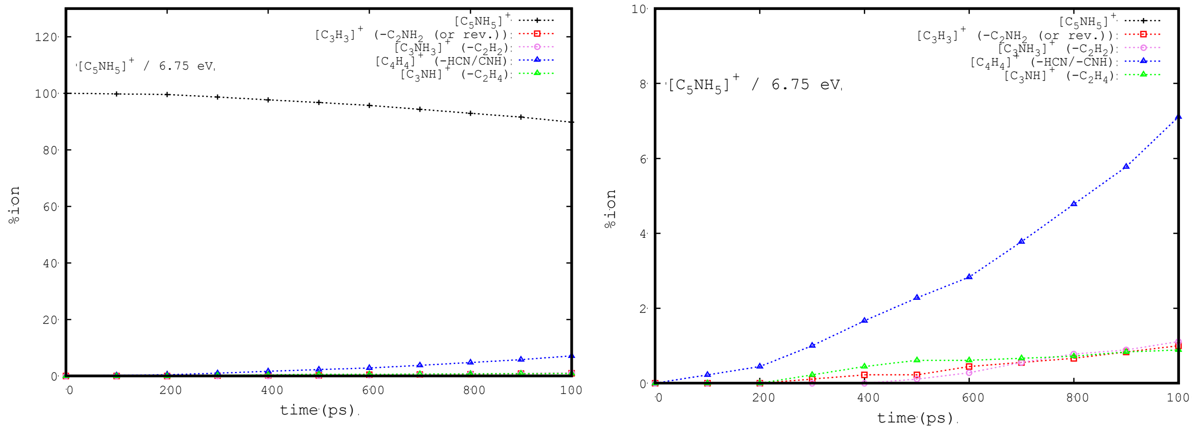

Supplementary Fig. 18: Kinetics for the dissociation of canonical pyridine$^{\bullet+}$ using Set1 parameters (1800 simulations at 6.74 eV internal energy of the respective cation).

Supplementary Tab. 1: Stoichiometry of the fragments resulting from the dissociation of benzonitrile$^{\bullet+}$ (pool of 1800 simulations) at the end of the MD/DFTB simulations for the two sets of parameters at different internal energies of the respective cation.

	**Set1 8.0 eV 1ns**	**Set1 8.22eV 700ps**	**Set1 8.5 eV 500 ps**	**Set1 8.8 eV 300 ps**	**Set2 8.0 eV 1 ns**	**Set2 8.35 eV 1 ns**
**$C_7NH_4^+$ (-H)**	**0**	**1**	**1**	**1**	**0**	**0**
**$C_6H_5^+$ (-CN)**	**0**	**0**	**0**	**1**	**0**	**0**
**$C_6H_4^+$ (-CNH/-HCN)**	**4 (3CNH, 1HCN) 3mBz/1oBz**	**10 (9CNH, 1HCN) 5mBz/1oBz/1l**	**20 (17CNH, 3 HCN)**	**15 (13CNH, 2HCN)**	**7 (4HCN, 3CNH) 6oBz,1lin**	**21 (12HCN, 9CNH)**
**$C_5NH_3^+$ (-C_2H_2)**	**0**	**1**	**1**	**0**	**1**	**5**
**C_5NH^+ (-C_2H_4)**	**0**	**1**	**3**	**1**	**0**	**3**
**$C_4H_4^+$ (-C_3NH)**	**0**	**2**	**0**	**2**	**4**	**10**
**$C_4H_2^+$ (-C_3NH_3)**	**1**	**1**	**0**	**3**	**2**	**5**
**Other fragments**			**1$C_5H_3^+$,1$C_4NH_2^+$ (or 3$C_3H_3^+$)**	**3$C_4NH_2^+$ (or 3$C_3H_3^+$)**	**1$C_4NH_2^+$,**	**1$C_5H_3^+$, 7$C_4NH_2^+$ (or 7$C_3H_3^+$)**

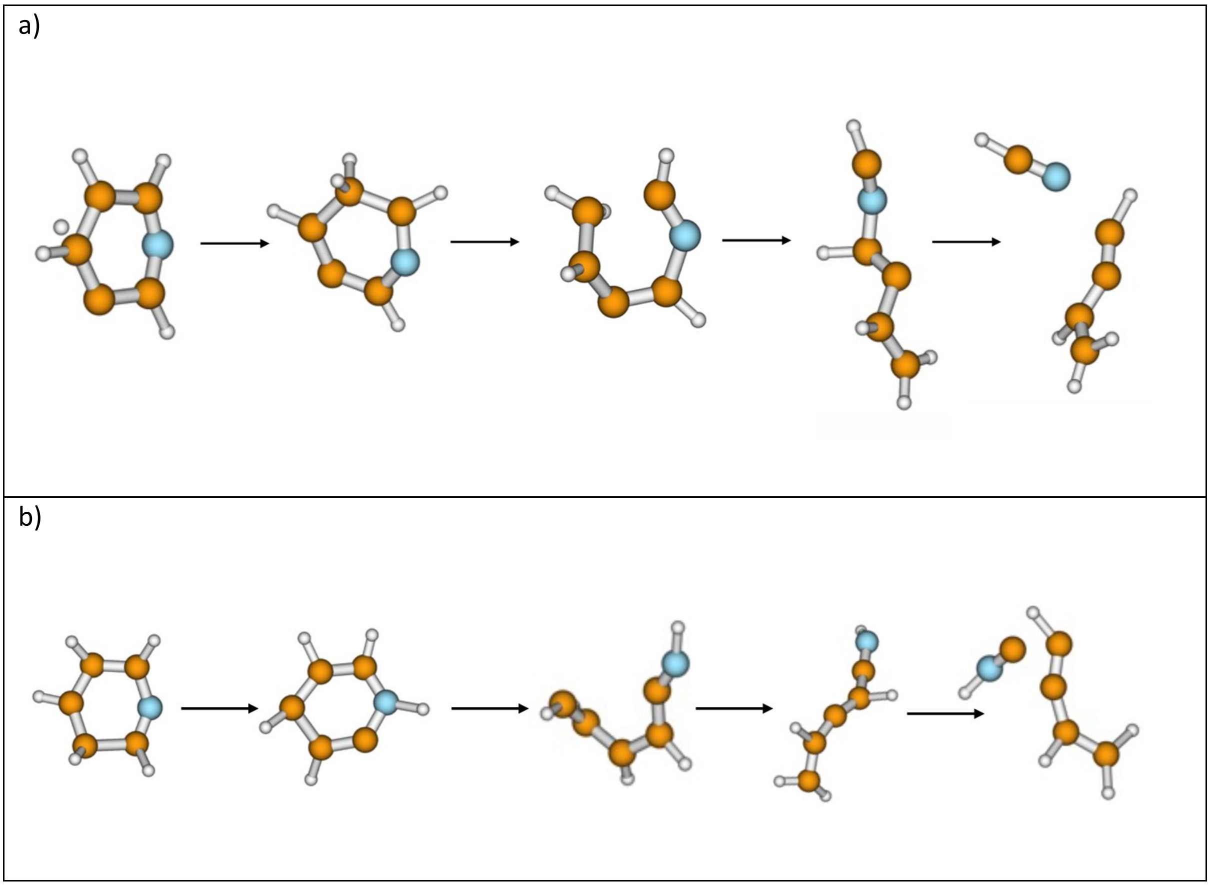

Supplementary Fig. 19: Exemplary pathways for loss of (a) HCN and (b) HNC from canonical pyridine$^{\bullet+}$ as observed in the MD/DFTB simulations at 6.74 eV internal energy of the respective cation using Set1 parameters.

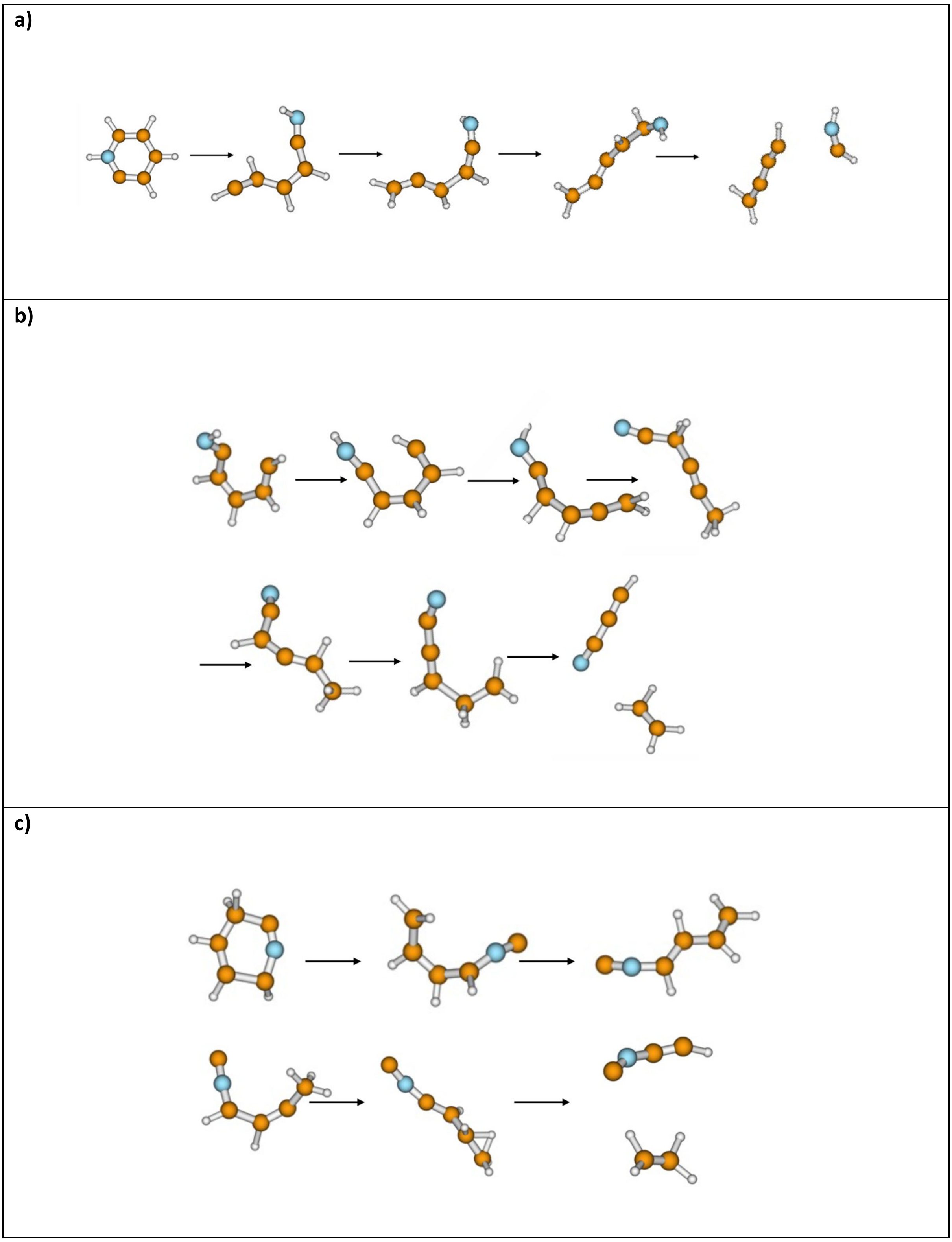

Supplementary Fig. 20: Exemplary pathways for the loss of (a) $H_2CN^{\bullet}$ and (b) C_2H_4 from the alpha-distonic pyridine$^{\bullet+}$ and loss of (c) C_2H_4 from the canonical pyridine$^{\bullet+}$ as observed in the MD/DFTB simulations 6.74 eV internal energy of the respective cation using Set1 parameters.

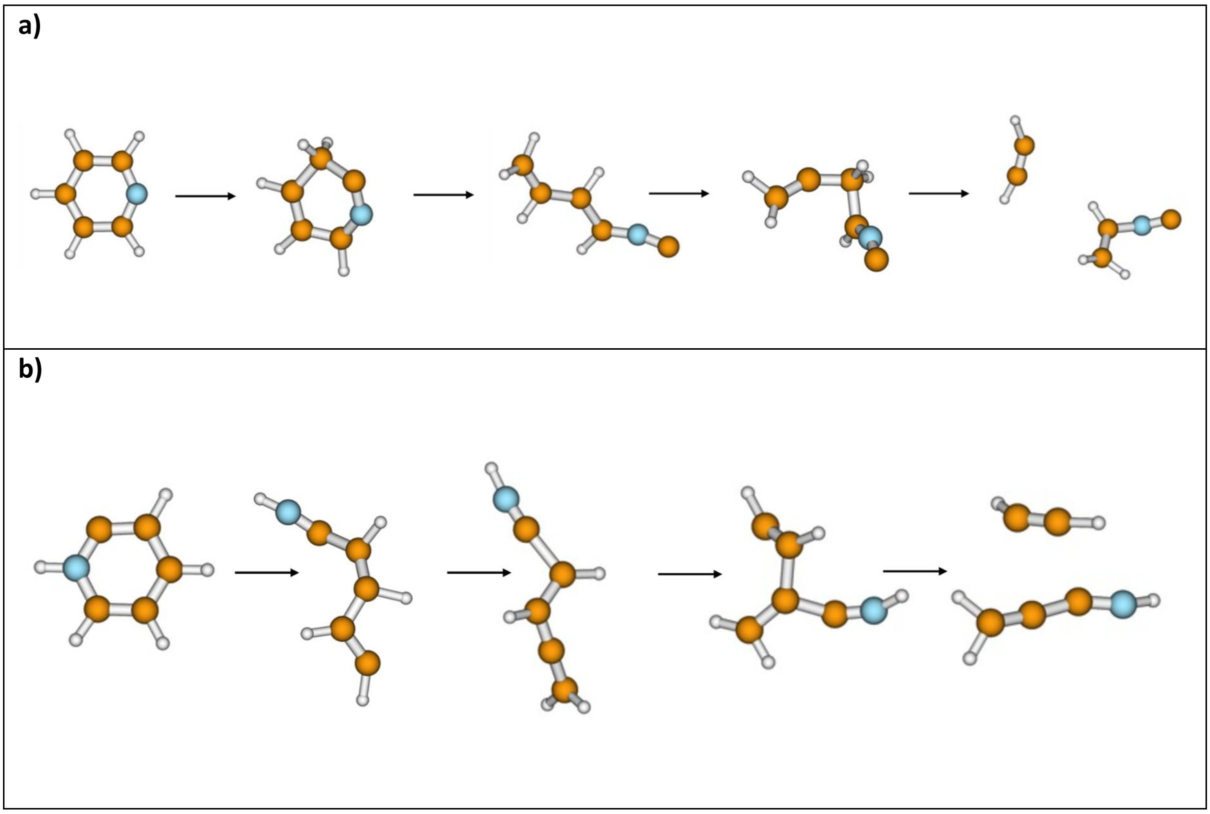

Supplementary Fig. 21: Exemplary pathways for the C_2H_2 loss from (a) canonical pyridine$^{\bullet+}$ and (b) alpha-distonic pyridine$^{\bullet+}$ ion as observed in the MD/DFTB simulations at 6.74 eV internal energy of the respective cation using Set1 parameters.

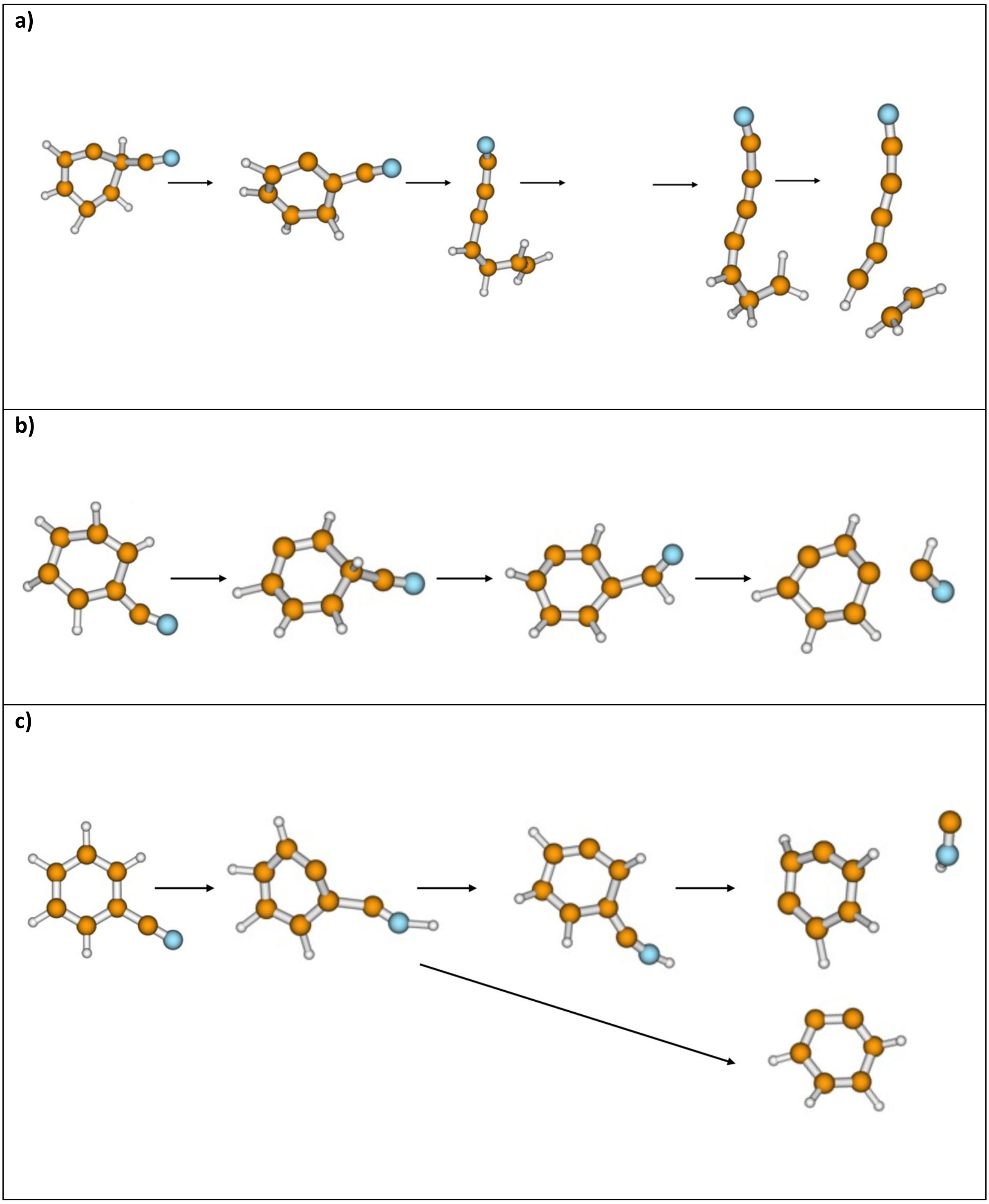

Supplementary Fig. 22: Exemplary pathways of C_2H_4 loss leading to (a) $HC_5N^{\bullet+}$, HCN loss leading to (b) m-benzyne$^{\bullet+}$ and HNC loss forming (c) m- and o-benzyne$^{\bullet+}$ from benzonitrile$^{\bullet+}$ as observed in the MD/DFTB simulations at 8.8 eV internal energy of the respective cation using Set1 parameters.

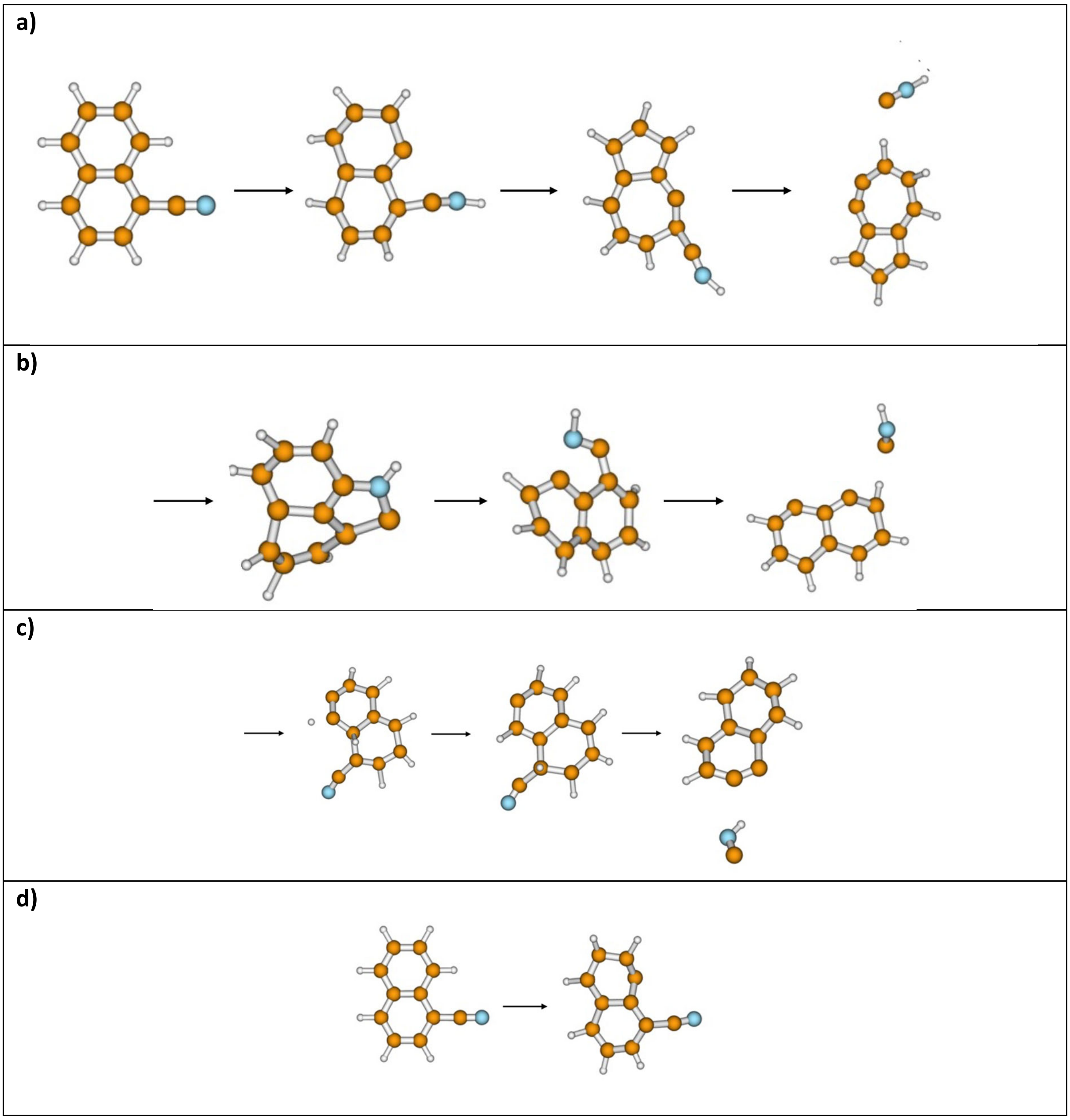

Supplementary Fig. 23: Exemplary pathways for the loss of (a-c) HNC as observed after 100 ps, displaying 3 different $C_{10}H_6^{\bullet+}$ isomers and (d) the direct H loss from 1-cyano-naphthalene$^{\bullet+}$ as observed in the MD/DFTB simulations at 12.7 eV internal energy of the respective cation using Set1 parameters.

Supplementary Tab. 2: Stoichiometry of the fragments resulting from the dissociation of 1-cyanonaphthalene$^{\bullet+}$ (pools of 720 simulations of 1 ns) obtained from MD/DFTB simulations at two internal energies of the respective cation.

Int. En. / Frag.	12.7 eV	13.6 eV
$C_{11}NH_6^+$ (-H)	1	8
$C_{10}H_7^+$ (-CN)	0	3
$C_{10}H_6^+$ (-HNC/-HCN)	93 (100%CNH)	227 (94%CNH, 6%HCN)
$C_9NH_4^+$ ($-C_2H_2$)	1	5
$C_4H_4^+$ ($-C_3NH$)	0	2
$C_4H_2^+$ ($-C_3NH_3$)	1	1

References

1 P. Jusko, S. Brünken, O. Asvany, S. Thorwirth, A. Stoffels, L. Van Der Meer, G. Berden, B. Redlich, J. Oomens and S. Schlemmer, *Faraday Discuss*, 2019, **217**, 172–202.

2 D. B. Rap, A. N. Marimuthu, B. Redlich and S. Brünken, *J Mol Spectrosc*, 2020, **373**, 111357.

3 D. B. Rap, J. G. M. Schrauwen, A. N. Marimuthu, B. Redlich and S. Brünken, *Nature Astronomy 2022 6:9*, 2022, **6**, 1059–1067.

4 D. Gerlich, *Inhomogeneous RF Fields: A Versatile Tool for the Study of Processes with Slow Ions*, John Wiley & Sons, Ltd, 2007.

5 D. Oepts, A. F. G. van der Meer and P. W. van Amersfoort, *Infrared Phys Technol*, 1995, **36**, 297–308.

6 A. N. Marimuthu, D. Sundelin, S. Thorwirth, B. Redlich, W. D. Geppert and S. Brünken, *J Mol Spectrosc*, 2020, **374**, 111377.

7 M. J. Frisch, G. W. Trucks, H. B. Schlegel, G. E. Scuseria, M. A. Robb, J. R. Cheeseman, G. Scalmani, V. Barone, G. A. Petersson, H. Nakatsuji, X. Li, M. Caricato, A. v. Marenich, J. Bloino, B. G. Janesko, R. Gomperts, B. Mennucci, H. P. Hratchian, J. v. Ortiz, A. F. Izmaylov, J. L. Sonnenberg, D. Williams-Young, F. Ding, F. Lipparini, F. Egidi, J. Goings, B. Peng, A. Petrone, T. Henderson, D. Ranasinghe, V. G. Zakrzewski, J. Gao, N. Rega, G. Zheng, W. Liang, M. Hada, M. Ehara, K. Toyota, R. Fukuda, J. Hasegawa, M. Ishida, T. Nakajima, Y. Honda, O. Kitao, H. Nakai, T. Vreven, K. Throssell, J. A. , Jr. Montgomery, J. E. Peralta, F. Ogliaro, M. J. Bearpark, J. J. Heyd, E. N. Brothers, K. N. Kudin, V. N. Staroverov, T. A. Keith, R. Kobayashi, J. Normand, K. Raghavachari, A. P. Rendell, J. C. Burant, S. S. Iyengar, J. Tomasi, M. Cossi, J. M. Millam, M. Klene, C. Adamo, R. Cammi, J. W. Ochterski, R. L. Martin, K. Morokuma, O. Farkas, J. B. Foresman and D. J. Fox, *Gaussian 16, Revision C.02*.

8 S. Panchagnula, J. Bouwman, D. B. Rap, P. Castellanos, A. Candian, C. Mackie, S. Banhatti, S. Brünken, H. Linnartz and A. G. G. M. G. M. Tielens, *Physical Chemistry Chemical Physics*, 2020, **22**, 21651–21663.

9 V. Barone, P. Cimino and E. Stendardo, *J Chem Theory Comput*, 2008, **4**, 751–764.

10 C. Puzzarini, M. Biczysko and V. Barone, *J Chem Theory Comput*, 2010, **6**, 828–838.

11 A. K. Lemmens, D. B. Rap, J. M. M. Thunnissen, C. J. Mackie, A. Candian, A. G. G. M. Tielens, A. M. Rijs and W. J. Buma, *Astron Astrophys*, 2019, **628**, A130.

12 T. Schwabe and S. Grimme, *Physical Chemistry Chemical Physics*, 2007, **9**, 3397–3406.

13 K. Steenbakkers, A. N. Marimuthu, B. Redlich, G. C. Groenenboom and S. Brünken, *J Chem Phys*, 2023, **158**, 084305.

14 B. Gans, S. Boyé-Péronne and J. Liévin, *J Chem Phys*, 2019, **150**, 244303.

15 M. Rapacioli, T. Heine, L. Dontot, M. Yusef Buey, F. Louisnard, J. Cuny, M. Morinière, C. Dubosq, S. Patchkovskii, J. Frenzel, E. Michoulier, H. Duarte, T. Minneva, F. Spiegelman, L. Zchekhov and D. Salahub, *2023 deMonNano experiment, https://demon-nano.ups-tlse.fr/*.

16 M. Elstner, D. Porezag, G. Jungnickel, J. Elsner, M. Haugk, T. Frauenheim, S. Suhai and G. Seifert, *Phys Rev B*, 1998, **58**, 7260–7268.

17 A. Simon, M. Rapacioli, G. Rouaut, G. Trinquier and F. X. Gadéa, *Philosophical Transactions of the Royal Society A: Mathematical, Physical and Engineering Sciences*, 2017, **375**, 20160195.

18 C. Joblin, G. Wenzel, S. Rodriguez Castillo, A. Simon, H. Sabbah, A. Bonnamy, D. Toublanc, G. Mulas, M. Ji, A. Giuliani and L. Nahon, *J Phys Conf Ser*, 2020, **1412**, 062002.

19 S. Banhatti, D. B. Rap, A. Simon, H. Leboucher, G. Wenzel, C. Joblin, B. Redlich, S. Schlemmer and S. Brünken, *Physical Chemistry Chemical Physics*, 2022, **24**, 27343–27354.

20 R. Flammang, M. Barbieux-Flammang, E. Gualano, P. Gerbaux, H. T. Le, M. T. Nguyen, F. Turecek and S. Vivekananda, *Journal of Physical Chemistry A*, 2001, **105**, 8579–8587.

21 L. Zheng, J. Cuny, S. Zamith, J. M. L'Hermite and M. Rapacioli, *Physical Chemistry Chemical Physics*, 2021, **23**, 27404–27416.

22 H. Leboucher, J. Mascetti, C. Aupetit, J. A. Noble and A. Simon, *Photochem*, 2022, **2**, 237–262.

23 T. L. Nguyen, J. H. Baraban, B. Ruscic and J. F. Stanton, *Journal of Physical Chemistry A*, 2015, **119**, 10929–10934.
